# Genetic interaction with temperature is an important determinant of nematode longevity

**DOI:** 10.1111/acel.12658

**Published:** 2017-09-21

**Authors:** Hillary Miller, Marissa Fletcher, Melissa Primitivo, Alison Leonard, George L. Sutphin, Nicholas Rintala, Matt Kaeberlein, Scott F. Leiser

**Affiliations:** ^1^ Cellular and Molecular Biology Program University of Michigan Ann Arbor MI 48109 USA; ^2^ Department of Pathology University of Washington Seattle WA 98195 USA; ^3^ Molecular & Integrative Physiology Department University of Michigan Ann Arbor MI 48109 USA; ^4^ Department of Internal Medicine University of Michigan Ann Arbor MI 48109 USA

**Keywords:** Aging, bacteria, *C. elegans*, Lifespan, Temperature

## Abstract

As in other poikilotherms, longevity in *C. elegans* varies inversely with temperature; worms are longer‐lived at lower temperatures. While this observation may seem intuitive based on thermodynamics, the molecular and genetic basis for this phenomenon is not well understood. Several recent reports have argued that lifespan changes across temperatures are genetically controlled by temperature‐specific gene regulation. Here, we provide data that both corroborate those studies and suggest that temperature‐specific longevity is more the rule than the exception. By measuring the lifespans of worms with single modifications reported to be important for longevity at 15, 20, or 25 °C, we find that the effect of each modification on lifespan is highly dependent on temperature. Our results suggest that genetics play a major role in temperature‐associated longevity and are consistent with the hypothesis that while aging in *C. elegans* is slowed by decreasing temperature, the major cause(s) of death may also be modified, leading to different genes and pathways becoming more or less important at different temperatures. These differential mechanisms of age‐related death are not unlike what is observed in humans, where environmental conditions lead to development of different diseases of aging.

## Introduction, Results, Discussion

The aging process has been described as stochastic—a probabilistic degeneration of cellular function that may be explained in sufficient detail by thermodynamic principles (Conti, 2008). Thermodynamics and the kinetics of chemical reactions provide the most rudimentary understanding of how physiological processes change as temperature changes. Described most simply, the rates of various chemical reactions increase as temperature increases, resulting in an increased rate of biochemical processes and, possibly, a corresponding increase in the rate of aging. Consistent with this model, lowering the ambient temperature of poikilotherms such as *C. elegans, D. melanogaster, and C. bellottii,* and decreasing a mouse's body temperature can increase lifespan (Loeb & Northrop, 1916; Lamb, 1968; Hosono *et al*., 1982; Conti *et al*., 2006).

**Figure 1:**
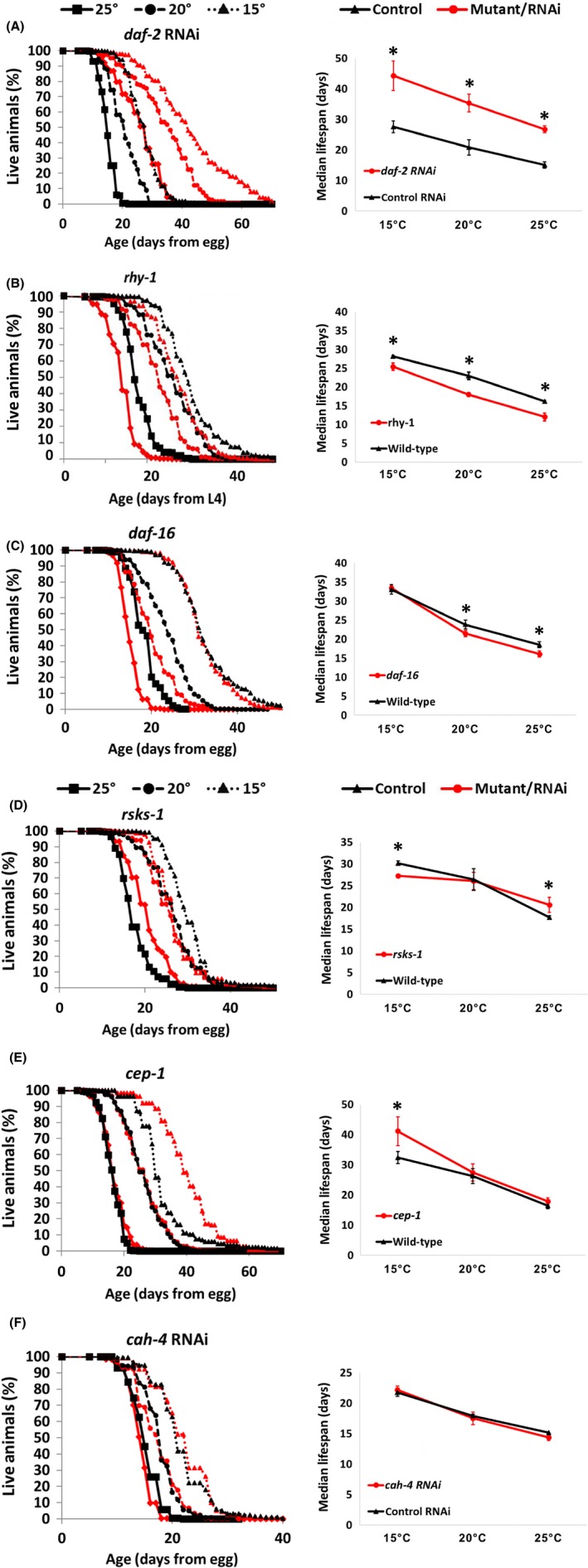
Examples of different types of interactions between genotype, temperature, and lifespan. (A–F) show survival curves and combined graphs plotting median lifespan vs temperature at 15°, 20°, and 25° for *daf‐2* (RNAi), *rhy‐1(ok1402), daf‐16(mu86), rsks‐1(ok1255)*,* cep‐1(gk138),* and *cah‐4* (RNAi) compared to wild‐type (N2). Note that because they are developmentally delayed, *rhy‐1* lifespans are shown from L4. All lifespans are available in Figs. S1–S3 (Supporting Information). Significant (P < .05) differences between control and experimental conditions denoted with asterisks (*).

In *C. elegans*, animals that develop and age at 15 °C (‘low temperature’) are long‐lived compared to wild‐type animals grown at 20 °C (~ room temperature), whereas wild‐type worms that develop and age at 25 °C (‘high temperature’) are short‐lived compared to wild‐type worms grown at 15 °C or 20 °C (Fig. 1). This ‘temperature law’ has been described as widely accepted, but not tested beyond limited number of strains (Zhang *et al*., 2015).

While the ‘temperature law’ is observed among wild‐type organisms, the interplay between genetics and temperature is not well understood. Multiple recent reports suggest that the effects of temperature on longevity are genetically controlled and that both heat and cold modify transcriptional pathways that effect lifespan (Lee & Kenyon, 2009; Leiser *et al*., 2011; Xiao *et al*., 2013; Ewald *et al*., 2015; Horikawa *et al*., 2015; Zhang *et al*., 2015; Chen *et al*., 2016). To better understand the interplay between temperature and longevity, we measured the lifespans of worms with genetic manipulations known to affect longevity at 15 °C, 20 °C, or 25 °C. Figure 1 illustrates six examples of how longevity can be impacted across temperatures, representing conditions that

- robustly increase lifespan at all temperatures (*daf‐2* RNAi)
- robustly decrease lifespan at all temperatures (*rhy‐1(ok1402)*)
- decrease lifespan at high but not low temperature (*daf‐16(mu86)*)
- increase lifespan at high temperature but decrease lifespan at low temperature (*rsks‐1(ok1255)*)
- increase lifespan at low temperature but not high temperature (*cep‐1(gk138)*)
- do not alter lifespan at any temperature (*cah‐4* RNAi)

**Figure 2:**
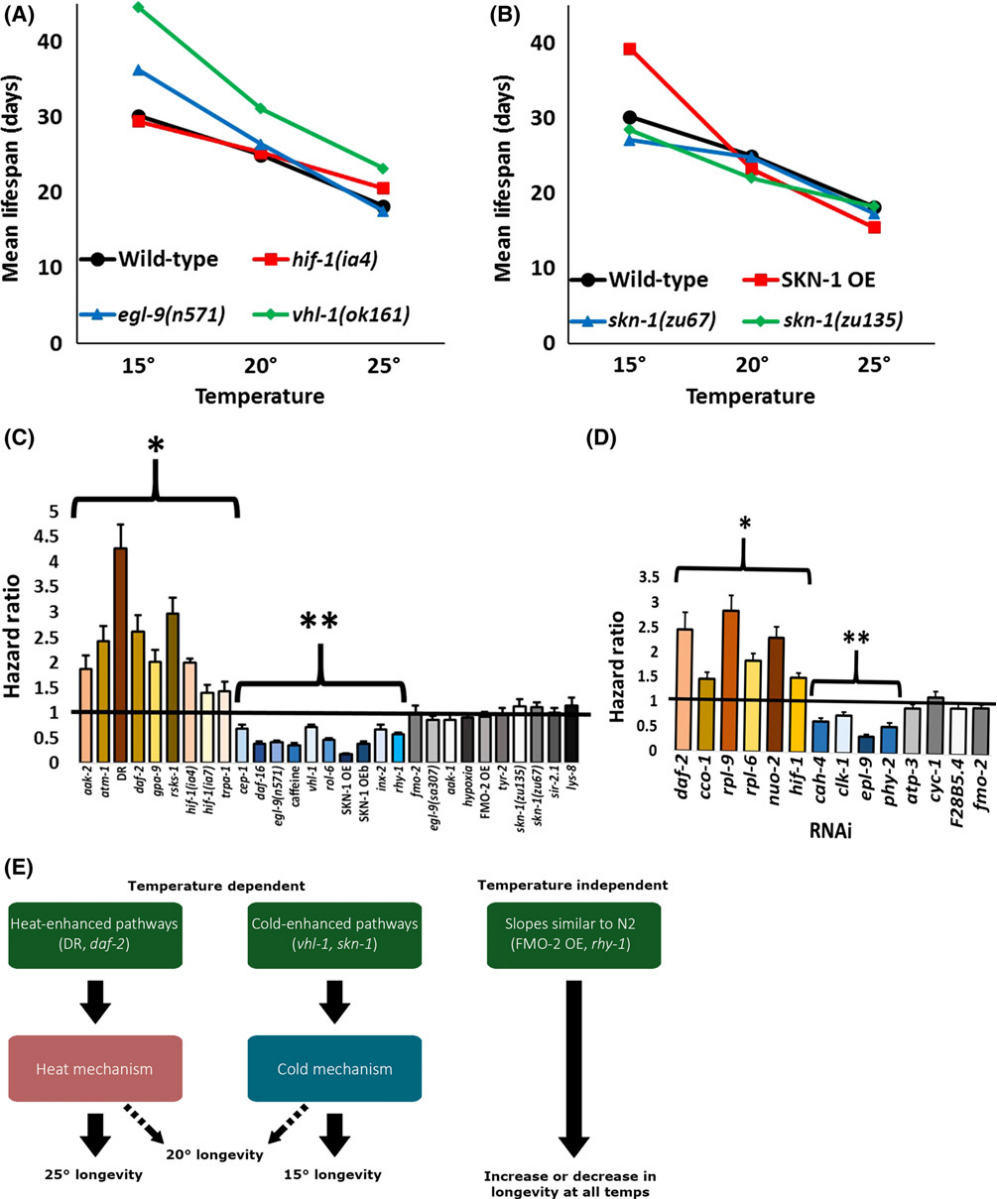
Temperature vs. longevity across genotypes. (A–B) plot median lifespan vs temperature at 15, 20, and 25 °C for opposing genetic conditions in the longevity pathways of hypoxic signaling and antioxidant signaling normalized to wild‐type (N2). (C–D) show the Cox regression‐calculated hazard ratios between each condition, separated into UV‐killed and RNAi conditions, across temperatures. (E) depicts a basic model. Significant (*P *< 0.01) increased (*) and decreased (**) hazard ratios at 15 °C compared to 25 °C are denoted.

Having established that relative longevity can vary across temperatures, we next asked whether this variability is common among conditions known to modify longevity. We tested nearly fifty genotypes and interventions previously reported to affect lifespan (Figs. S1–S3 and Tables S1 and S2) and found that relative longevity was consistently inconsistent across temperatures (Fig. S4). However, there are consistent trends within longevity pathways, where strains/conditions known to have opposing effects are also affected by temperature oppositely (Fig.  2A,B, Fig.  S5A–D). We used Cox regression analysis to assess the interaction between each longevity intervention and temperature. The hazard ratios, which represent the cumulative risk of death throughout a worm's lifespan, confirm the interaction between condition (genotype, RNAi, etc.) and temperature and clearly separate the conditions into three categories: approximately one‐third (15/43) of the interventions show an increased hazard ratio (significantly ‘better’ at higher temperature), one‐third (14/43) show a decreased hazard ratio (significantly ‘better’ at lower temperature), and one‐third (14/43) show no interaction between genetic manipulation and temperature (Fig. 2C,D). The changes in hazard ratio are frequently ~twofold and are clearly not random, as evidenced by reciprocal results for genes that are known to have opposite effects within the same pathway (e.g., *daf‐2(e1370)* vs. *daf‐16(mu86)*,* vhl‐1(ok161)* vs. *hif‐1(ia4)*) (Fig.  S6). Figure S7 provides a heat map analysis with hierarchical clustering that segregates into the groups described in Figure 1.

In summary, we find significant interaction between longevity interventions and environmental temperature in two‐thirds (29/43) of the cases examined, indicating that a temperature‐independent effect on longevity is more the exception than the rule (Fig. 2C,D). This variation confirms that genetics play a substantive role in temperature‐dependent longevity that cannot be explained solely by the rules of thermodynamics and chemical kinetics.

The observed variation in relative longevity with temperature is consistent with the hypothesis that distinct mechanisms determine nematode longevity at different temperatures (Fig. 2E). As shown in the model, there are three distinct types of strains/conditions: those with similar slopes and hazard ratios to N2 (FMO‐2 OE, *rhy‐1(ok1402)*, etc.), ‘temperature‐dependent’ strains/conditions that live comparatively longer at higher temperatures (e.g., DR, *rsks‐1* (*ok1255)*,* daf‐2(e1370),* or RNAi), and ‘temperature‐dependent’ strains/conditions that live comparatively longer at colder temperatures (*vhl‐1(ok161)*,* cep‐1(gk138)*, SKN‐1 OE). These three categories are further complicated by how they compare to wild‐type overall, leading some strains to be consistently long‐lived (e.g., *daf‐2(e1370)* or RNAi) or short‐lived (e.g., *rhy‐1 (ok1402)*), whereas other strains vary in relative longevity depending on temperature (e.g., *cep‐1(gk138)*). Together, these results suggest that testing strains/conditions at multiple temperature will not only define the robustness of an effect, but may provide clues as to the mechanism.

It has been suggested that protein quality control and the heat stress response are of primary importance for determining nematode longevity at 25 °C (Seo *et al*., 2013). Our data support this model; we find interventions that limit heat stress response (e.g., *daf‐16(mu86)*) are detrimental at high, but not low, temperature, while interventions that improve protein homeostasis, such as dietary restriction or reduced expression of translation machinery (e.g., *rsks‐1(ok1255), rpl‐6* RNAi), show lifespan extension at high temperature. The relevant mechanisms affecting longevity at low temperature are less clear, particularly because relatively few aging studies are conducted at 15 °C compared to 20 °C or 25 °C. It is possible a combination of a strain's ability to avoid age‐associated vulval integrity defects (AVID), a healthspan phenotype primarily observed at colder temperatures (Leiser *et al*., 2016), and to better adapt to temperature‐dependent changes to their bacterial food source (growth rate, metabolism, pathogenicity), leads to better outcomes in colder temperatures. We note that a subset of our data (*trpa‐1(ok999), daf‐16(mu86)* at 15 °C) differ from other published works on whether strains are relatively short or long‐lived at a given temperature (Xiao *et al*., 2013; Horikawa *et al*., 2015). While we did not directly test why these differences are observed, we expect that they are due to our lifespans using UV‐killed bacteria for a food source and others using live bacteria. It is known that *daf‐16* plays an important role in immunity (Singh & Aballay, 2009) in worms and both Xiao *et al*. and Chen *et al*.'s reports describe a requirement for *daf‐16* in their pathway. Our results agree with these reports on the slopes of the lifespans of these strains, and the differences we observe are consistent with immunity being more important at lower temperature. The differences between studies are similar to differences between live and UV‐killed food experiments (which live longer) (Garigan *et al*., 2002), and are worth exploring in future studies as they may explain cold‐dependent longevity mechanisms of insulin and *trpa‐1(ok999)* signaling.

Our results demonstrate that the impact of temperature on relative lifespan is of greater importance than generally appreciated by the *C. elegans* aging field. The vast majority of published studies report the impact of different interventions on lifespan at a single temperature, usually either 20 °C or 25 °C. We suggest that studies reporting effects on lifespan should typically be performed at more than one temperature to understand the robustness of the effect and the interaction with temperature. As further mechanistic studies on the factors that control differences in the relative lifespan vs. temperature axis are completed, we expect that plausible links will be made between temperature‐specific longevity in nematodes and specific diseases of aging in mammals.

## Conflict of Interest

The authors declare no conflict of interest.

## Funding

National Institutes of Health, (Grant/Award Number: ‘R00AG045200’, 'R01AG038518','T32AG000057','T32GM007315') National Institute of Aging, (Grant/Award Number: ‘P30AG024824’).

## Supporting information


**Fig. S1** Lifespans from L4 for strains with developmental delays.
**Fig. S2** Mutant and environmental condition lifespans at 15, 20, and 25 °C.
**Fig. S3** RNAi lifespans at 15, 20, and 25 °C.
**Fig. S4** Complete graph of median lifespan vs temperature at 15, 20, and 25 °C for all lifespan data normalized to wild‐type/control.
**Fig. S5** Pathway specific lifespans across temperatures by mean lifespan.
**Fig. S6** Cox regression‐calculated hazard ratios between each condition and wild‐type across temperatures (25‐15 °C) for the pathways described in Fig S5.
**Fig. S7** Heat map of relative longevity
**Table S1.** Descriptions of the 43 conditions included in. Figs S1 and S2.
**Table S2.** Lifespan information for Figs. 1, 2, S1, and S2.
**Table S3.** Hazard Ratio calculations for Fig. 2C‐D, Fig. S6.
