# Supplementary material for: Genetic interaction with temperature is an important determinant of nematode longevity
Source: Aging Cell. 2017 Sep 21;16(6):1425–9. doi: 10.1111/acel.12658 (PMC5676069; doi:10.1111/acel.12658)
Supplement: Supplementary file 1 — Fig. S1 Lifespans from L4 for strains with developmental delays. Fig. S2 Mutant and environmental condition lifespans at 15, 20, and 25 °C. Fig. S3 RNAi lifespans at 15, 20, and 25 °C. Fig. S4 Complete graph of median lifespan vs temperature at 15, 20, and 25 °C for all lifespan data normalized to wild‐type/control. Fig. S5 Pathway specific lifespans across temperatures by mean lifespan. Fig. S6 Cox regression‐calculated hazard ratios between each condition and wild‐type across temperatures (25‐15 °C) for the pathways described in Fig S5. Fig. S7 Heat map of relative longevity Table S1. Descriptions of the 43 conditions included in. Figs S1 and S2. Table S2. Lifespan information for Figs. 1, 2, S1, and S2. Table S3. Hazard Ratio calculations for Fig. 2C‐D, Fig. S6. [file ACEL-16-1425-s001.docx]

**Supplemental Material**

**Materials and Methods**

**Strains and Growth Conditions**

Standard procedures for *C. elegans* strain maintenance (wormbook.org) were used where experiments were performed on animals fed UV-killed Escherichia coli (OP50) from egg and maintained on solid nematode growth medium (NGM) with 50 μg/mL ampicillin added. Supplementary Table 1 includes a list of the strains and RNAi conditions used in this study.

**Lifespan measurements**

Lifespans were carried out as previously described with minor modifications (Sutphin and Kaeberlein 2009). Briefly, 20 - 25 Gravid adult animals were placed on NGM plates for a timed egg-lay. After 8-16 hours, depending on the temperature, these animals were removed. Once their progeny reached late L4/early adult stage, animals were transferred to plates with 33 µL of 150 mM fluorodeoxyuridine (FUdR) and 100 µL of 50 mg/mL Ampicillin per 100 mL NGM to prevent the development of progeny and growth of bacteria. Roughly 75 worms were placed on each NGM + FUdR plate seeded with concentration (20×) UV-killed OP50. A minimum of two plates per strain per condition were used per replicate. Lifespan plates were transferred periodically during early adulthood to prevent starvation and avoid contamination. Animals were scored as dead and removed from the experiment when they did not move in response to prodding under a dissection microscope. Temperature sensitive (ts) alleles, (e.g. daf-2(e1370)) were housed at 20° then moved to 25° after development, along with their wild-type controls.

**RNAi knockdown**

The RNAi feeding bacteria were obtained from the Vidal RNAi library. All RNAi plasmids were sequenced to verify the correct target sequence. Animals were exposed to RNAi plates from egg on plates consisting of NGM supplemented with 1 mM β-D-isothiogalactopyranoside (IPTG) and 25 μg/ml carbenicillin. At late L4 stage of development the animals were transferred to plates containing freshly seeded RNAi bacteria plus FUdR.

**Hypoxia treatment**

We followed a protocol as previously described (Fawcett, Horsman, and Miller 2012) to design hypoxia flow chambers to continuously house animals throughout their life at .5% oxygen after development and placement on FUdR.

**Dietary restriction (DR) treatment**

DR assays were performed like other lifespans until the second (25°C), third (20°C), or fourth (15°C) day of adulthood, when the worms were transferred to plates without a bacterial food source. This form of DR is termed bacterial deprivation (BD) (Kaeberlein et al. 2006). Since complete removal of all food causes a fleeing response in worms, we added 100uL of 10mM palmitic acid to the outer rim of the plate to prevent flight.

**Caffeine treatment**

Solid anhydrous caffeine was added directly to the NGM solution prior to autoclaving and pouring as previously described (Sutphin et al. 2012). Neither autoclaving nor UV treatment during plate preparation influenced life span in the presence of caffeine.

**Statistical analyses**

Lifespan data and statistics are included in Supplementary Table 2. Supplementary Table 3 provides the results of Cox proportional hazards regression models, which were run in Stata 14. The model includes a categorical variable for temperature, using 25 degrees as the base category, and including two dummy variables for 15 and 20 degrees. It also includes a dummy variable for experimental versus control. The main variables of interest for this paper are the interactions between the experimental dummy and the temperature dummies, which capture the differential effect of temperature on experimental versus control worms. Hierarchical clustering of the differences between conditions and control median lifespan across temperatures was performed using the heatmap.2 function in the R package “Gplots”. These values were calculated by subtracting the control animal’s median age at death from the condition’s median age at death then dividing by the control animal’s mean age at death. All error bars shown in figures represent the standard error of the mean.

**References for Methods Section:**

Fawcett, E. M., J. W. Horsman, and D. L. Miller. 2012. "Creating defined gaseous environments to study the effects of hypoxia on C. elegans." *J Vis Exp* (65):e4088. doi: 10.3791/4088.

Kaeberlein, T. L., E. D. Smith, M. Tsuchiya, K. L. Welton, J. H. Thomas, S. Fields, B. K. Kennedy, and M. Kaeberlein. 2006. "Lifespan extension in Caenorhabditis elegans by complete removal of food." *Aging Cell* 5 (6):487-94.

Sutphin, G. L., E. Bishop, M. E. Yanos, R. M. Moller, and M. Kaeberlein. 2012. "Caffeine extends life span, improves healthspan, and delays age-associated pathology in Caenorhabditis elegans." *Longev Healthspan* 1:9. doi: 10.1186/2046-2395-1-9.

Sutphin, G. L., and M. Kaeberlein. 2009. "Measuring Caenorhabditis elegans life span on solid media." *J Vis Exp* (27). doi: 1152 [pii] 10.3791/1152.

**Supplemental Figure 1. Lifespans from L4 for strains with developmental delays.**

| **A** | **B** | **C** |
| --- | --- | --- |
| **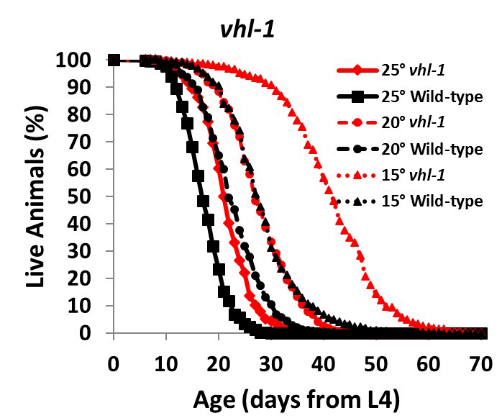** | **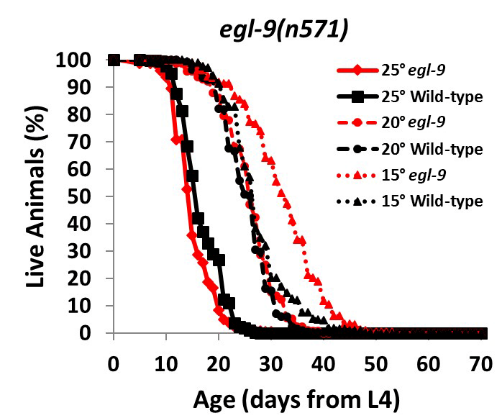** | **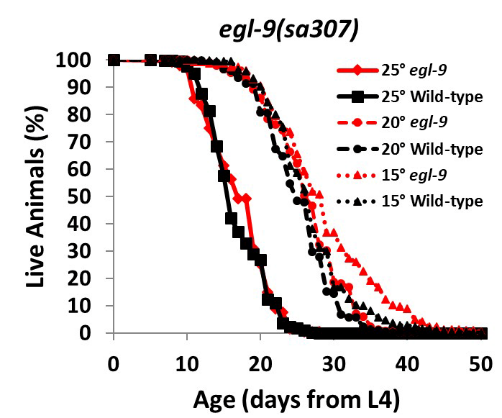** |
| **D** | **E** |  |
| **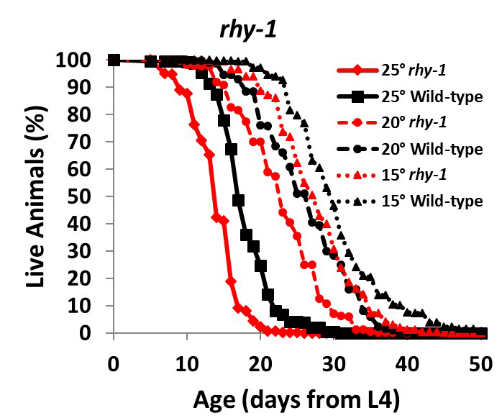** | **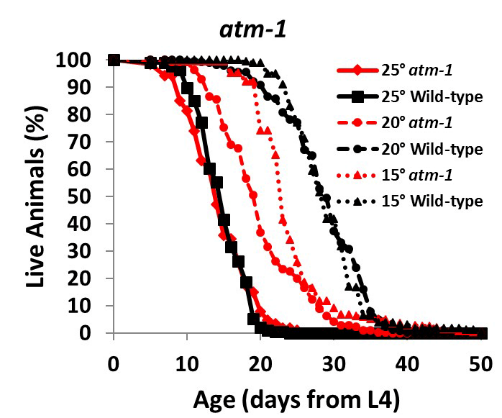** |  |

| **Supplemental Figure 2. Mutant and environmental condition lifespans at 15, 20, and 25°C.** | | |
| --- | --- | --- |
| **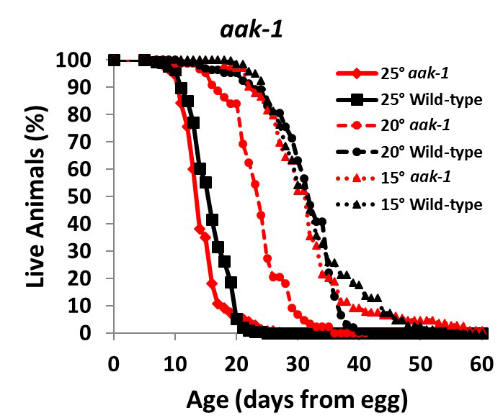** | **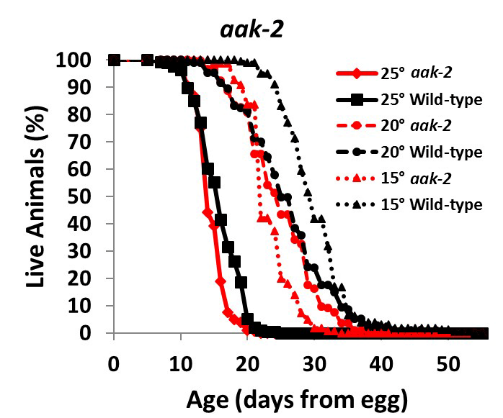** | **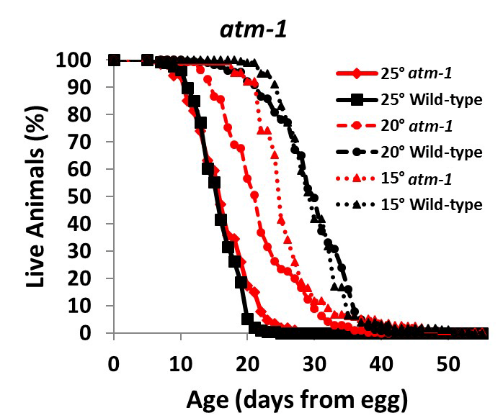** |
| **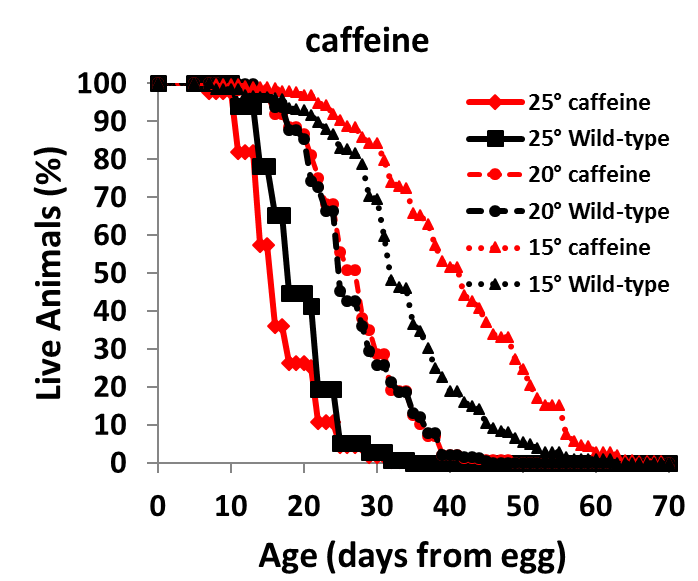** | **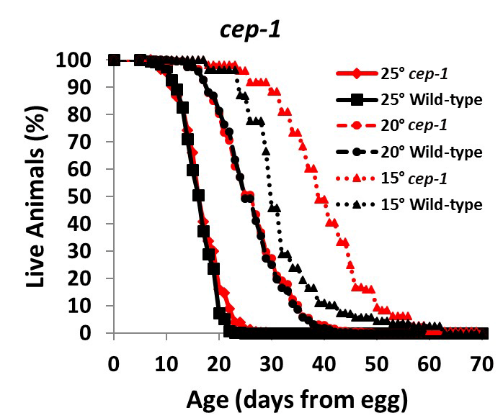** | **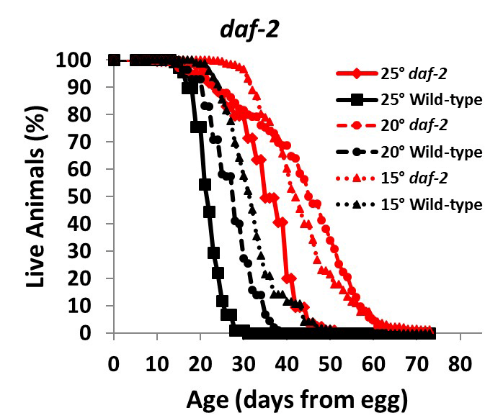** |
| **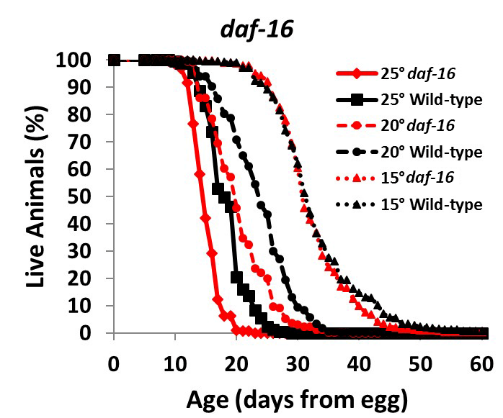** | **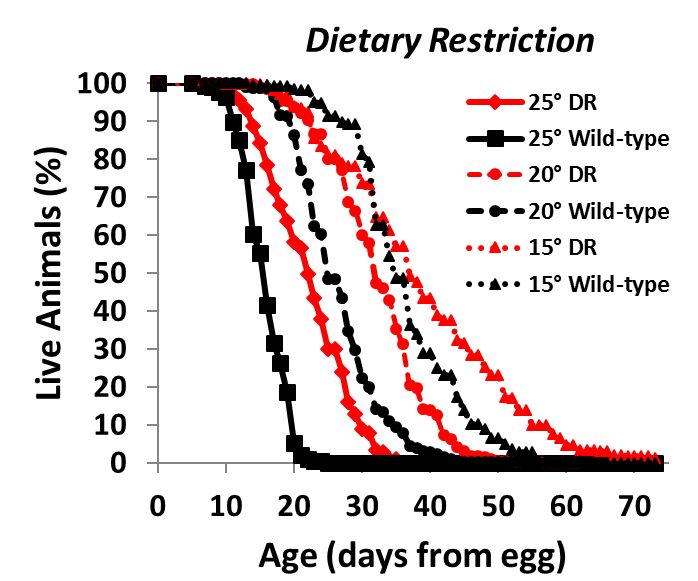** | **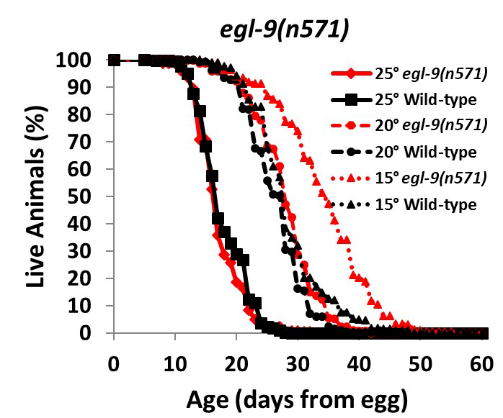** |
| **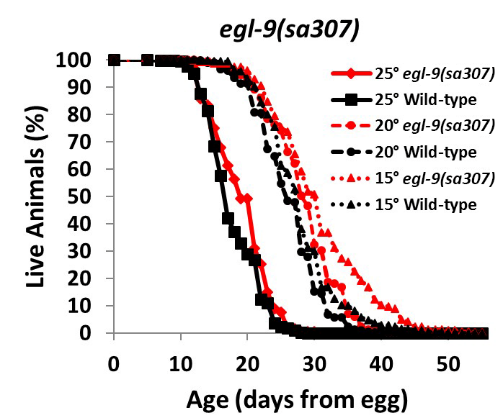** | **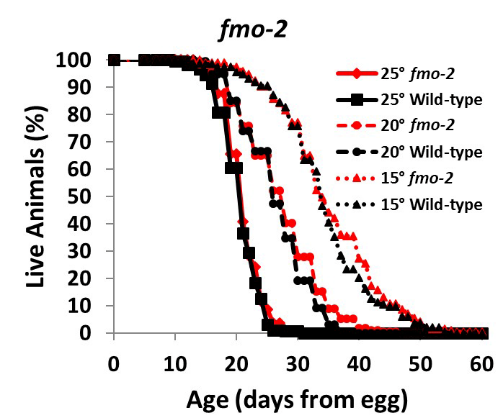** | **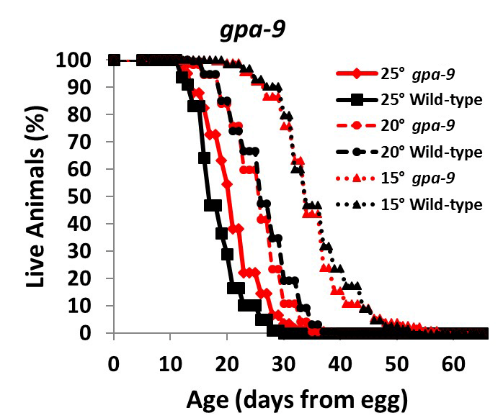** |
| **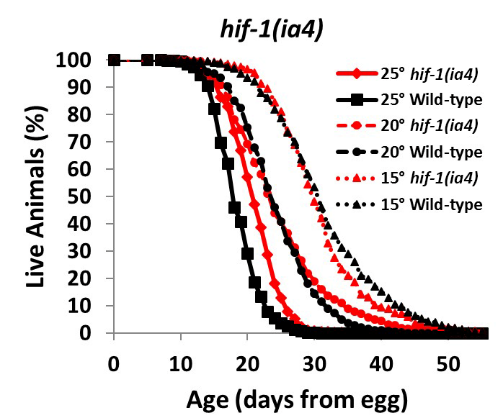** | **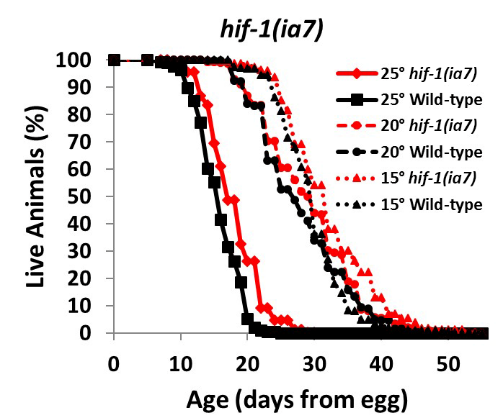** | **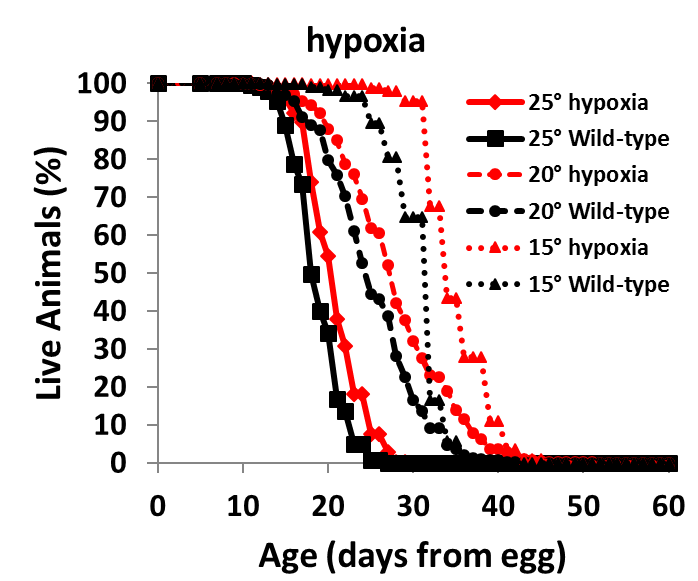** |
| **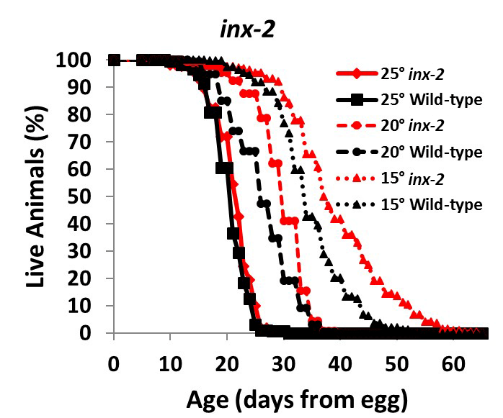** | **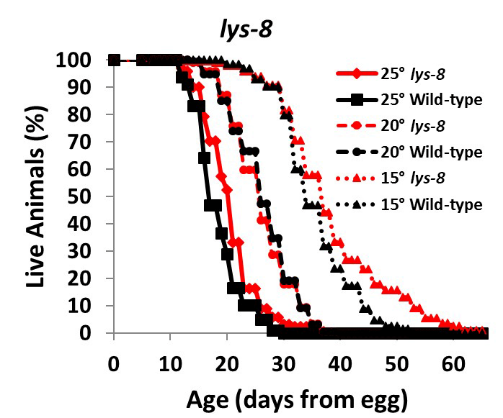** | **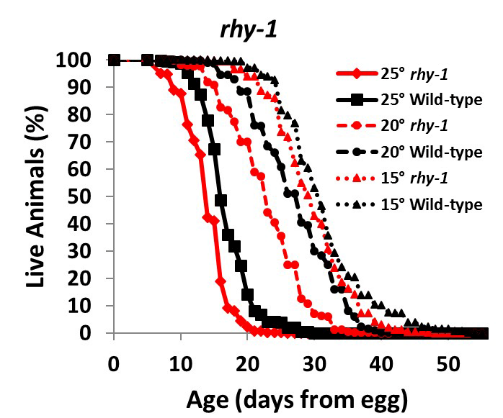** |
| **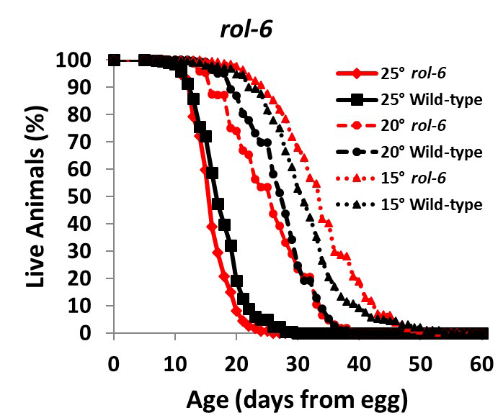** | **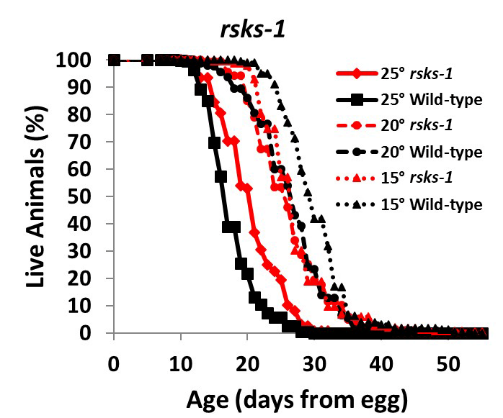** | **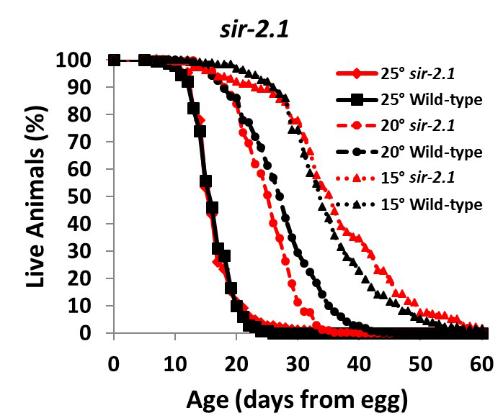** |
| **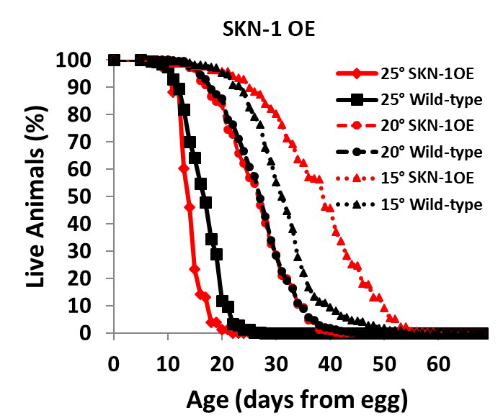** | **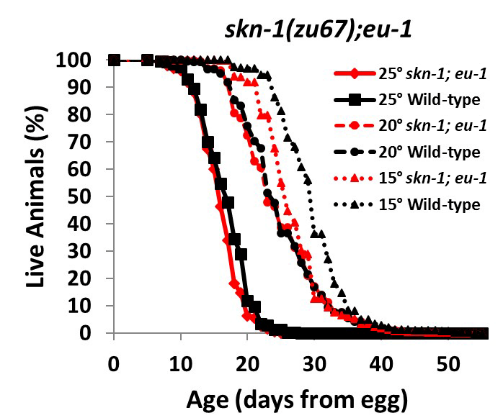** | **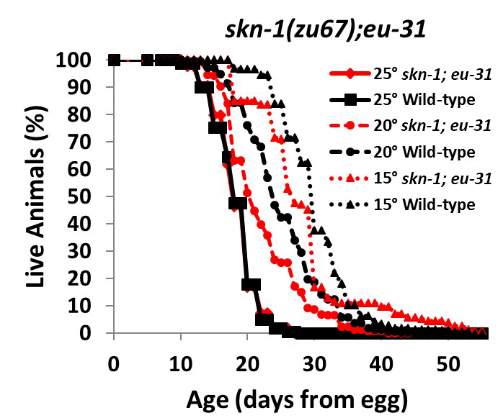** |
| **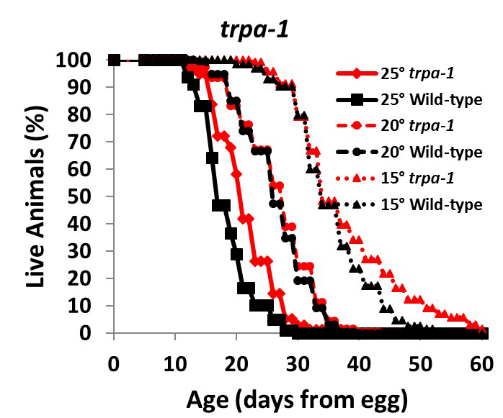** | **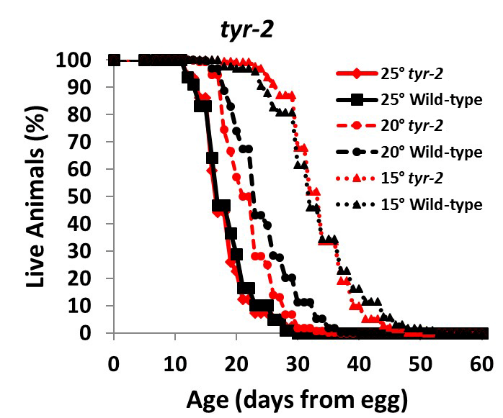** | **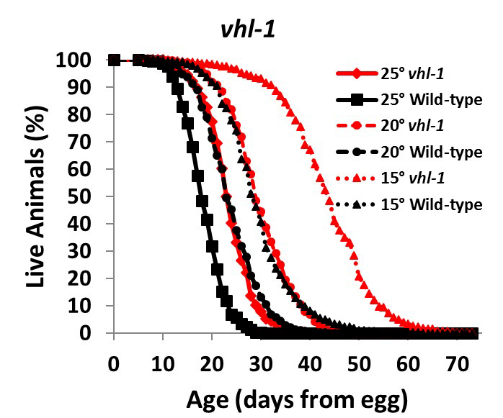** |

| **Supplemental Figure 3. RNAi lifespans at 15, 20, and 25°C.** | | |
| --- | --- | --- |
| **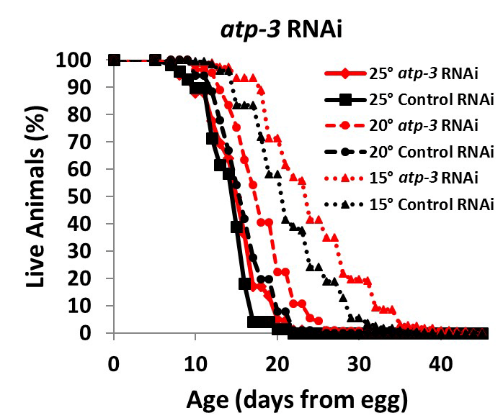** | **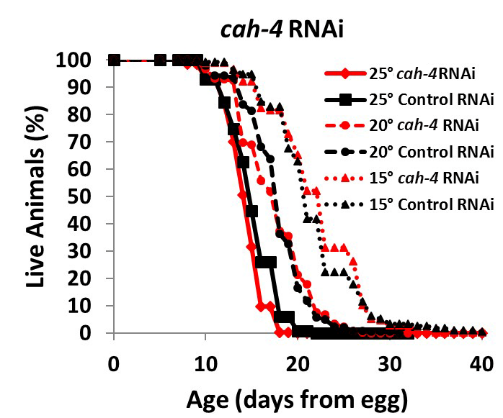** | **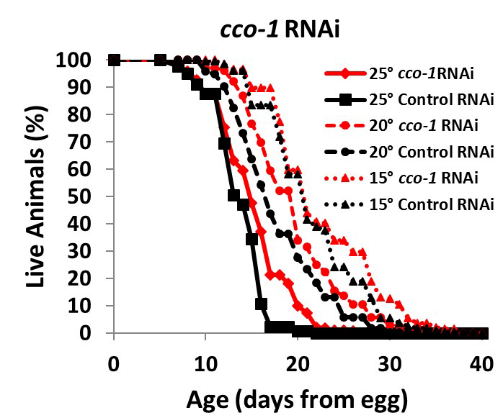** |
| **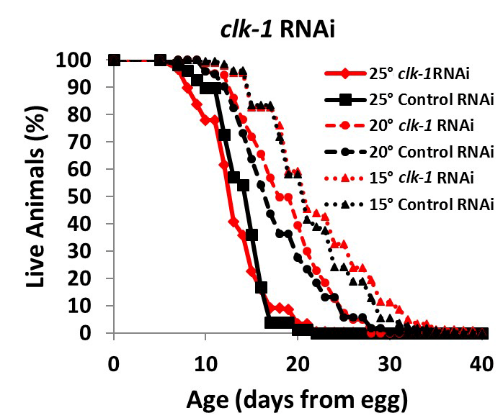** | **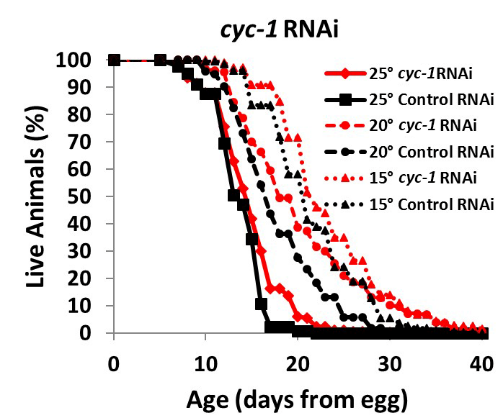** | **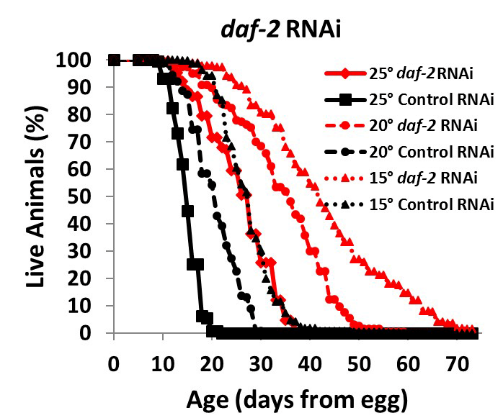** |
| **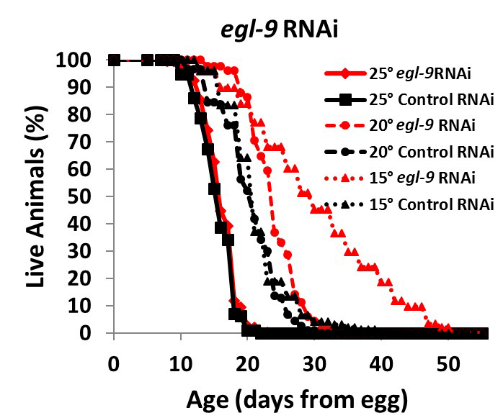** | **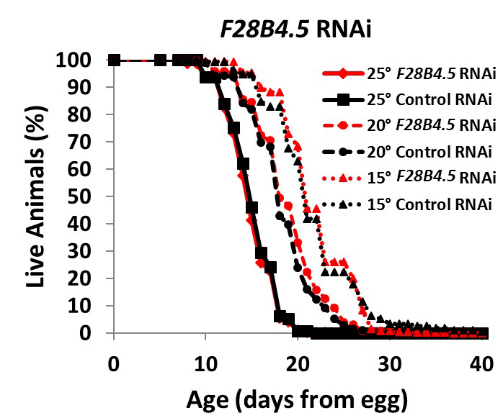** | **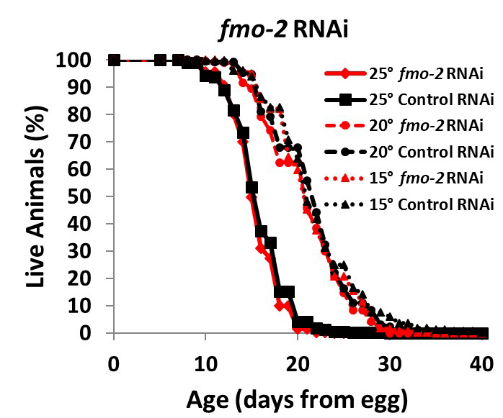** |
| **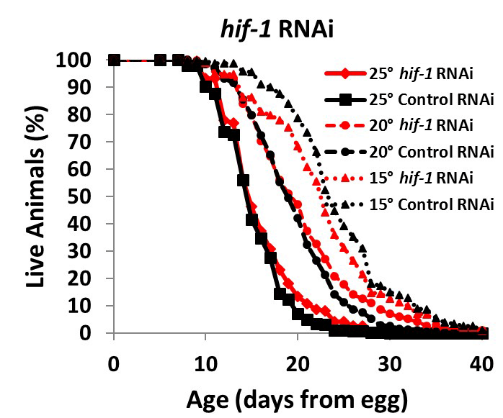** | **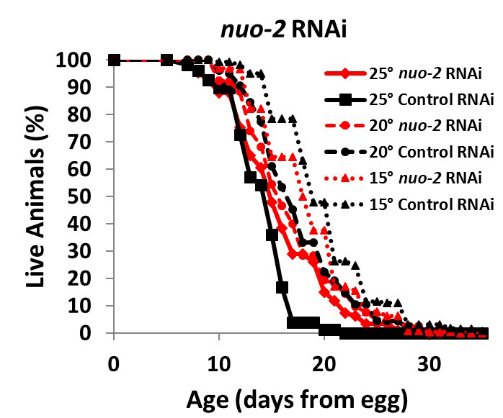** | **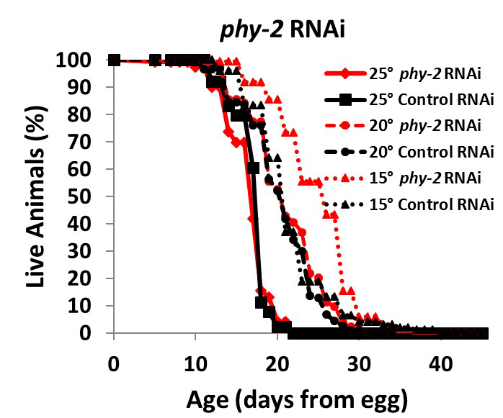** |
| **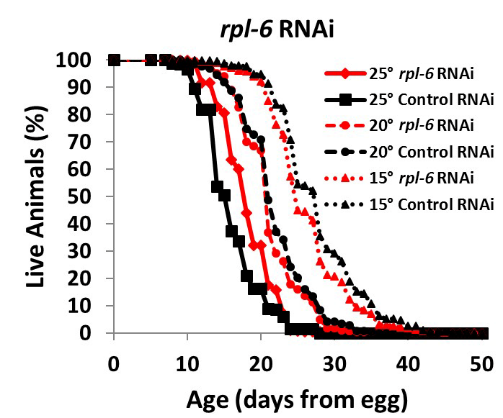** | **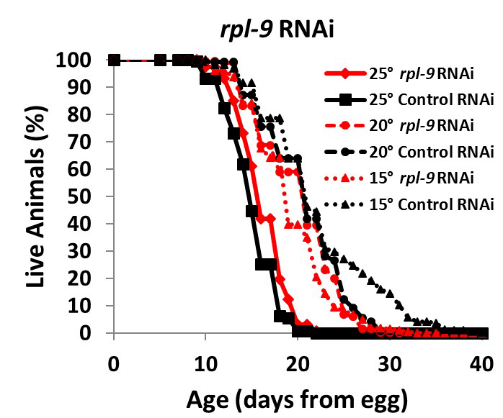** | **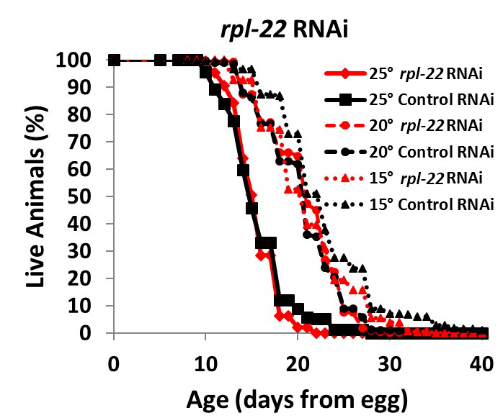** |

**Supplemental Figure 4. Complete graph of median lifespan vs temperature at 15, 20, and 25°C for all lifespan data normalized to wild-type/control.**

| **A** |
| --- |
| **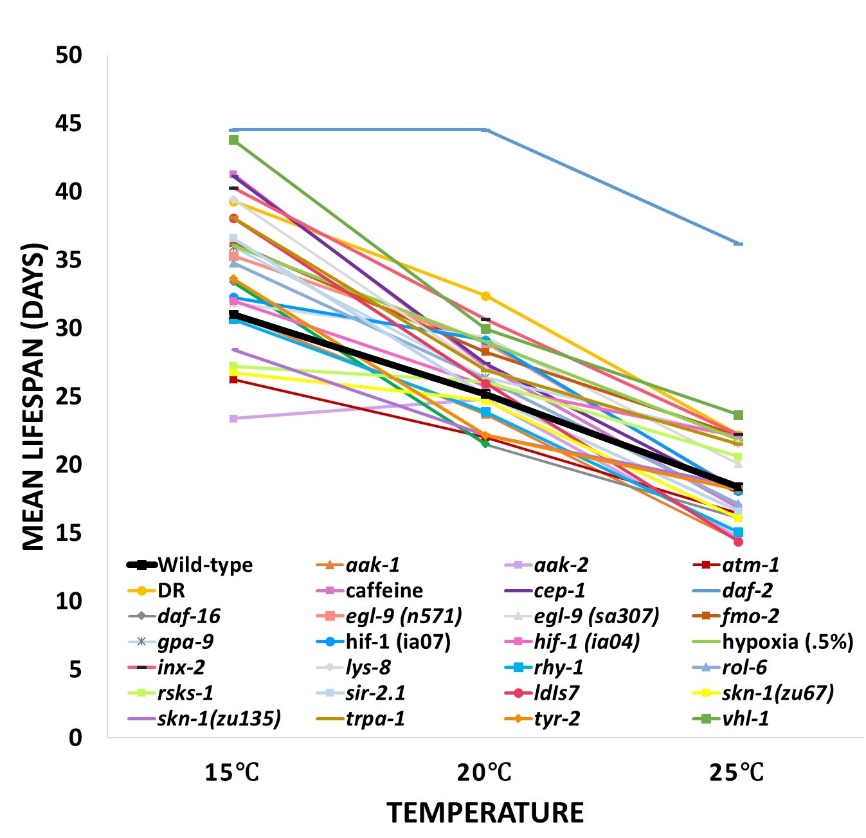** |
| **B** |
| **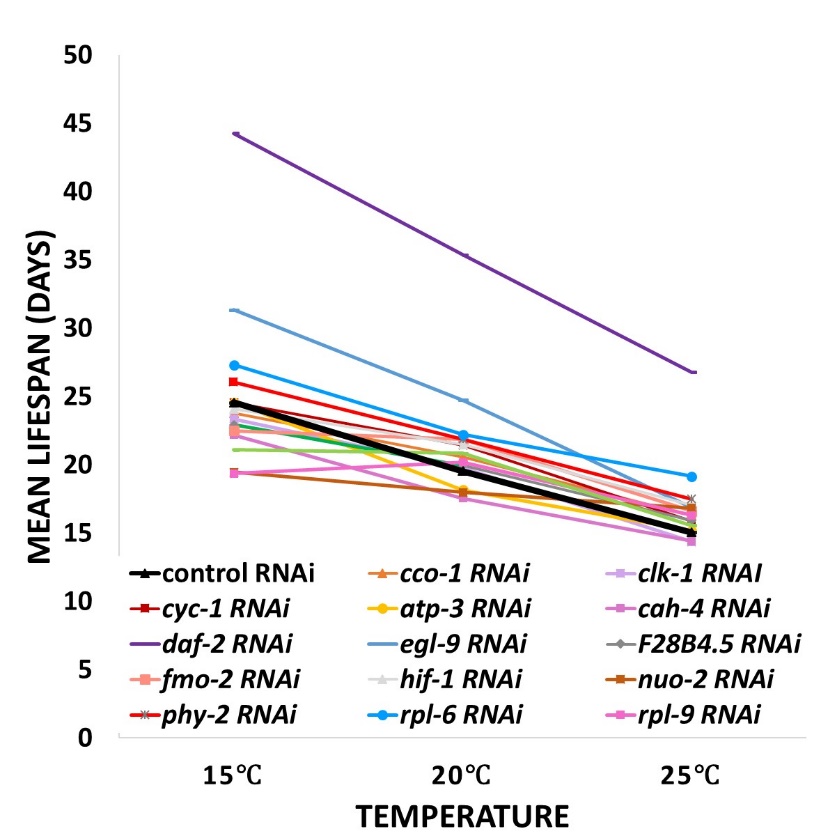** |

**Supplemental Figure 5. Pathway specific lifespans across temperatures by mean lifespan.**

| **A**  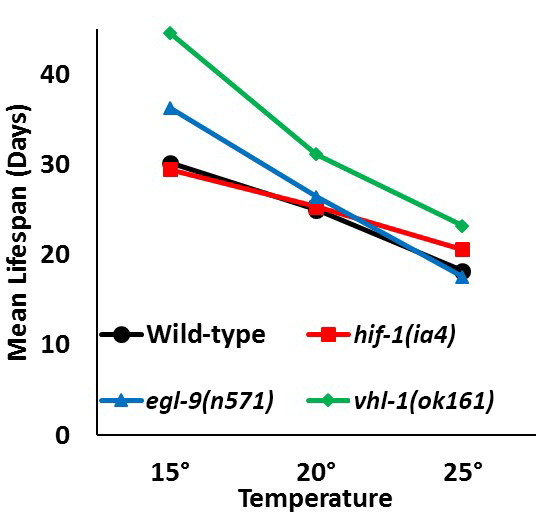 | **B**  **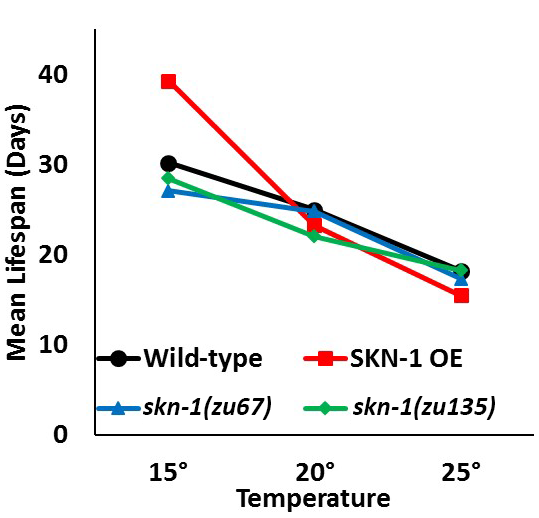** |
| --- | --- |
| **C**  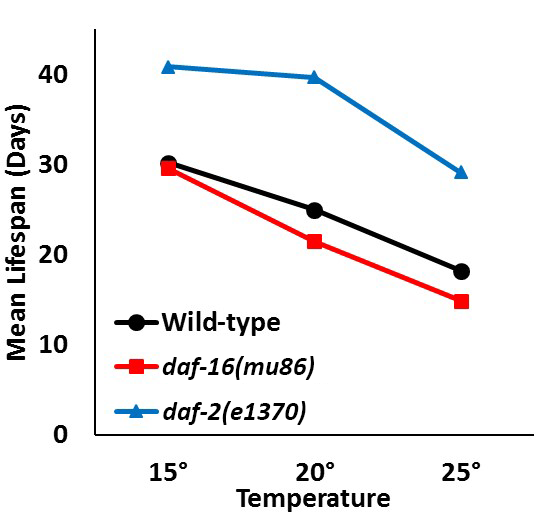 | **D**  **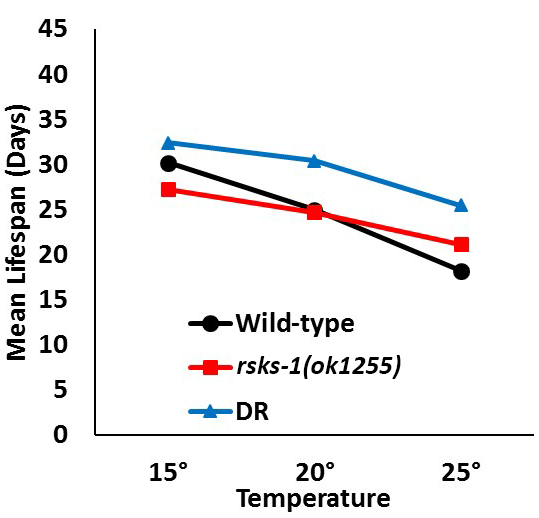** |

**Supplemental Figure 5. Pathway specific lifespans across temperatures by mean lifespan.**

Panels A-D plot median lifespan vs temperature at 15, 20, and 25°C for opposing genetic conditions in the longevity pathways of, hypoxic signaling (A), antioxidant signaling (B), insulin signaling (C), and dietary restriction/mTOR (D) normalized to wild-type (N2).

**Supplemental Figure 6. Cox regression-calculated hazard ratios between each condition and wild-type across temperatures (25-15°C) for the pathways described in Figure S5.**

| **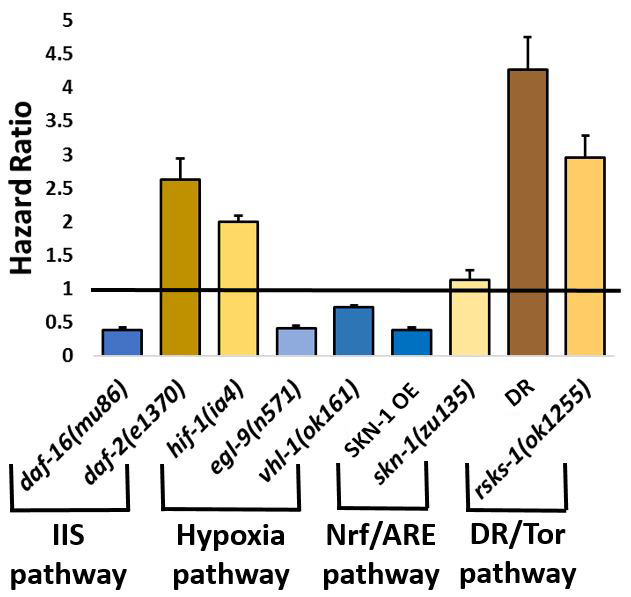** |
| --- |
|  |

**Supplemental Figure 7. Heat map of relative longevity.**

|  |
| --- |
| 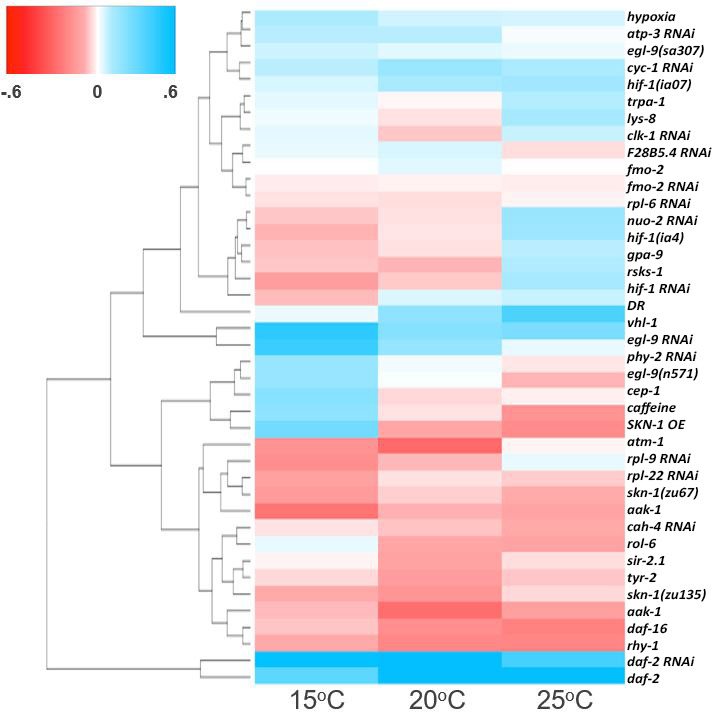 |

**Supplemental Figure 7. Heatmap of relative longevity.**

A clustered heatmap where red shading depicts longer average lifespan than wild-type and blue shading depicts a shorter average lifespan than wild-type.

**Supplemental Table 1. Descriptions of the 43 conditions included in supp. Figures 1 and 2.**

| **Condition** | **Strain/Dose** | **Description** |
| --- | --- | --- |
| **aak-1** | AGD397 | AMPK, downstream of insulin signaling (DAF-16 activation) and energy sensing |
| **aak-2** | RB754 | AMPK, downstream of insulin signaling (DAF-16 activation) and energy sensing |
| **atm-1** | VC381 | ATM, DNA damage response |
| **atp-3** | RNAi | ATP synthase, regulates mitochondrial ATP production |
| **dietary restriction** | bacteria deprivation | Robust intervention to enhance longevity |
| **caffeine** | 10mM | Commonly used compound, previously reported to be long-lived |
| **cah-4** | RNAi | Carbonic anhydrase, downstream of hypoxic response (HIF-1 activation) |
| **cco-1** | RNAi | Cytochrome c oxidase, regulates mitochondrial respiration |
| **cep-1** | MN1 | p53 ortholog, downstream multiple longevity pathways |
| **clk-1** | RNAi | COQ7/CAT5 ortholog, regulates mitochondrial respiration |
| **cyc-1** | RNAi | Cytochrome c reductase, regulates mitochondrial respiration |
| **daf-2** | CB1370 | Insulin-like receptor, negative regulator of DAF-16 |
| **daf-2** | RNAi | Insulin-like receptor, negative regulator of DAF-16 |
| **daf-16** | CF1038 | FOXO homologue, transcription factor downstream of insulin signaling |
| **egl-9** | MT1201 | Proline hydroxylase, negative regulator of HIF-1 |
| **egl-9** | JT307 | Proline hydroxylase, negative regulator of HIF-1 |
| **egl-9** | RNAi | Proline hydroxylase, negative regulator of HIF-1 |
| **F28B4.5** | RNAi | Unknown function, downstream of hypoxic response (HIF-1 activation) |
| **fmo-2** | VC1668 | Detoxification enzyme, downstream of hypoxic response (HIF-1 activation) |
| **fmo-2** | RNAi | Detoxification enzyme, downstream of hypoxic response (HIF-1 activation) |
| **gpa-9** | NL793 | GTPase, involved in innate immunity |
| **hif-1** | ZG31 | Hypoxia inducible factor, transcription factor activated by low oxygen (< 1%) |
| **hif-1** | ZG596 | Hypoxia inducible factor, transcription factor activated by low oxygen (< 1%) |
| **hif-1** | RNAi | Hypoxia inducible factor, transcription factor activated by low oxygen (< 1%) |
| **hypoxia** | .5% | Intervention to activate HIF-1 |
| **inx-2** | CX13325 | Innexin/gap junction protein, downstream of hypoxic response (HIF-1 activation) |
| **lys-8** | RB2528 | Lysozyme, involved in innate immunity |
| **nuo-2** | RNAi | NDUFS3 ortholog, regulates mitochondrial respiration |
| **phy-2** | RNAi | Downstream of the hypoxic response (HIF-1 activation) |
| **rhy-1** | RB1297 | Negative regulator of HIF-1 |
| **rol-6** | CB187 | Collagen protein, common selection marker for transgenic animals |
| **rpl-6** | RNAi | Ribosomal subunit L6, inhibits mRNA translation (improves proteostasis) |
| **rpl-9** | RNAi | Ribosomal subunit L9, inhibits mRNA translation (improves proteostasis) |
| **rpl-22** | RNAi | Ribosomal subunit L22, inhibits mRNA translation (improves proteostasis) |
| **rsks-1** | RB1206 | Ribosomal S6 kinase, inhibits mRNA translation (improves proteostasis) |
| **sir-2.1** | VC199 | NAD-deacetylase, reported to be important for longevity |
| **SKN-1 OE** | LD1 | Nrf ortholog, antioxidant response, transcription factor activated by ROS |
| **skn-1** | EU1 | Nrf ortholog, antioxidant response, transcription factor activated by ROS |
| **skn-1** | EU31 | Nrf ortholog, antioxidant response, transcription factor activated by ROS |
| **trpa-1** | RB1052 | TRP channel, detects changes in temperature |
| **tyr-2** | RB1272 | Tyrosinase ortholog, downstream of hypoxic response (HIF-1 activation) |
| **vhl-1** | CB5602 | E3 ubiquitin ligase, negative regulator of HIF-1 |

**Supplemental Table 2. Lifespan information for Figures 1, 2, S1, and S2.**

|  | **15oC** |  |  |  |  |  |  |  | |
| --- | --- | --- | --- | --- | --- | --- | --- | --- | --- |
| **Genotype/**  **Treatment** | **Averaged Mean** | **Exp. Error** | **Rep** | **n** | **Total Mean** | **Total Error** | **Total Median** | **p-value (compared to N2)** |  |
| **aak-1 (tm1944)** | 31.1164 | 1.4499 | 3 | 277 | 31.805 | 0.4826 | 32.00 | 0.000575 |  |
| **N2 (control)** | 32.3977 | 2.7532 |  | 350 | 32.817 | 0.3840 | 32.00 |  |  |
| **aak-2 (ok524)** | 23.7323 | 0.7995 | 2 | 166 | 23.385 | 0.2544 | 22.00 | 3.1E-35 |  |
| **N2 (control)** | 29.7591 | 1.3617 |  | 227 | 30.185 | 0.3346 | 30.00 |  |  |
| **atm-1 (gk186)** | 26.4768 | 1.9071 | 2 | 283 | 26.254 | 0.3479 | 25.00 | 4.45E-12 |  |
| **N2 (control)** | 29.7591 | 1.3617 |  | 227 | 30.185 | 0.3346 | 30.00 |  |  |
| **atp-3 (RNAi)** | 23.8828 | 1.3261 | 3 | 322 | 24.555 | 0.3482 | 24.55 | 1.9E-16 |  |
|  | 21.9532 | 2.3897 |  | 427 | 21.686 | 0.2526 | 21.69 |  |  |
| **Dietary Restriction (DR)** | 38.6467 | 2.9079 | 4 | 608 | 39.355 | 0.5305 | 39.35 | 1.19E-16 |  |
| **Fed (control)** | 37.1172 | 1.1827 |  | 432 | 36.641 | 0.3858 | 36.64 |  |  |
| **10mM caffeine** | 39.5129 | 2.2892 | 11 | 670 | 41.286 | 0.4421 | 41.28 | 1.95E-14 |  |
| **Water (control)** | 33.8995 | 1.4479 |  | 754 | 33.836 | 0.3582 | 33.84 |  |  |
| **cah-4 (RNAi)** | 22.0451 | 0.3965 | 3 | 325 | 22.181 | 0.2704 | 22.18 | 0.465556 |  |
| **vector (control)** | 21.8685 | 0.2140 |  | 484 | 21.793 | 0.2223 | 21.79 |  |  |
| **cco-1 (RNAi)** | 22.9238 | 2.0090 | 3 | 390 | 22.833 | 0.2960 | 22.83 | 0.000166 |  |
| **vector (control)** | 21.9532 | 2.3897 |  | 427 | 21.686 | 0.2526 | 21.69 |  |  |
| **cep-1 (gk138)** | 40.9166 | 4.6852 | 2 | 237 | 40.303 | 0.5739 | 39.00 | 8.56E-19 |  |
| **N2 (control)** | 33.1881 | 3.9937 |  | 243 | 32.449 | 0.5669 | 30.00 |  |  |
| **clk-1 (RNAi)** | 22.7224 | 2.3020 | 3 | 329 | 22.431 | 0.3314 | 22.43 | 1.46E-05 |  |
| **vector (control)** | 21.9532 | 2.3897 |  | 427 | 21.686 | 0.2526 | 21.69 |  |  |
| **cyc-1 (RNAi)** | 23.6866 | 0.7375 | 3 | 325 | 23.600 | 0.3425 | 23.60 | 0.001109 |  |
| **vector (control)** | 21.9532 | 2.3897 |  | 427 | 21.686 | 0.2526 | 21.69 |  |  |
| **daf-2 (e1370)** | 43.3146 | 1.3712 | 3 | 354 | 43.641 | 0.5002 | 42.00 | 8.91E-36 |  |
| **N2 (control)** | 32.0260 | 2.3993 |  | 336 | 32.253 | 0.3546 | 32.00 |  |  |
| **daf-2 (RNAi)** | 44.3884 | 4.6223 | 4 | 383 | 43.396 | 0.7026 | 43.39 | 2.51E-35 |  |
| **vector (control)** | 27.9567 | 1.6142 |  | 474 | 27.553 | 0.2579 | 27.55 |  |  |
| **daf-16 (mu86)** | 32.3871 | 0.9495 | 7 | 899 | 32.450 | 0.2057 | 31.00 | 5.89E-07 |  |
| **N2 (control)** | 32.8469 | 1.4034 |  | 775 | 33.089 | 0.2692 | 32.00 |  |  |
| **egl-9 (n571)** | 35.0808 | 1.9758 | 4 | 544 | 34.373 | 0.3185 | 35.00 | 6.21E-11 |  |
| **N2 (control)** | 28.7036 | 0.8776 |  | 550 | 28.573 | 0.2511 | 28.00 |  |  |
| **egl-9 (RNAi)** | 30.4919 | 0.0361 | 2 | 236 | 30.483 | 0.6498 | 30.48 | 1.28E-20 |  |
| **vector (control)** | 21.6729 | 0.1503 |  | 380 | 21.666 | 0.2537 | 21.67 |  |  |
| **egl-9 (sa307)** | 31.7281 | 2.2265 | 5 | 602 | 30.895 | 0.2924 | 31.00 | 0.000296 |  |
| **N2 (control)** | 28.3366 | 1.6297 |  | 586 | 27.894 | 0.2320 | 28.00 |  |  |
| **F28B4.5 (RNAi)** | 22.0068 | 0.3252 | 3 | 371 | 21.991 | 0.2058 | 21.99 | 0.93052 |  |
| **vector (control)** | 21.8685 | 0.2140 |  | 484 | 21.793 | 0.2223 | 21.79 |  |  |
| **fmo-2 (RNAi)** | 21.9147 | 1.3001 | 4 | 456 | 21.535 | 0.1972 | 21.53 | 0.000694 |  |
| **vector (control)** | 22.3478 | 1.2185 |  | 655 | 22.119 | 0.1916 | 22.12 |  |  |
| **fmo-2 (ok2147)** | 35.6377 | 0.4584 | 2 | 197 | 35.421 | 0.5869 | 34.00 | 0.442727 |  |
| **N2 (control)** | 34.4879 | 1.4879 |  | 247 | 34.494 | 0.4974 | 34.00 |  |  |
| **gpa-9 (pk438)** | 35.0242 | 0.8438 | 2 | 277 | 35.057 | 0.4069 | 34.00 | 0.003043 |  |
| **N2 (control)** | 35.8475 | 1.8273 |  | 271 | 35.679 | 0.3971 | 34.00 |  |  |
| **hif-1 (ia07)** | 32.5907 | 0.8500 | 3 | 383 | 32.258 | 0.3409 | 32.25 | 0.001283 |  |
| **N2 (control)** | 29.5709 | 0.8084 |  | 371 | 29.801 | 0.2660 | 29.80 |  |  |
| **hif-1 (ia04)** | 31.2084 | 0.6691 | 19 | 2446 | 31.036 | 0.1437 | 31.03 | 1.5E-11 |  |
| **N2 (control)** | 32.5583 | 1.1201 |  | 2282 | 31.784 | 0.1717 | 31.78 |  |  |
| **hif-1 (RNAi)** | 23.9526 | 1.6006 | 10 | 1097 | 23.096 | 0.2000 | 23.09 | 2.14E-20 |  |
| **vector (control)** | 25.2758 | 1.4691 |  | 1070 | 24.782 | 0.1852 | 24.78 |  |  |
| **hypoxia (.5%)** | 35.1186 | 0.1878 | 2 | 329 | 35.158 | 0.2089 | 34.00 | 1.57E-13 |  |
| **normoxia (21%)** | 30.9734 | 1.2266 |  | 368 | 30.647 | 0.1788 | 32.00 |  |  |
| **inx-2 (ok376)** | 39.3706 | 0.8091 | 3 | 406 | 39.384 | 0.4461 | 37.50 | 6.21E-09 |  |
| **N2 (control)** | 34.8984 | 1.4191 |  | 394 | 34.843 | 0.3282 | 34.00 |  |  |
| **lys-8 (ok3504)** | 38.7615 | 0.4712 | 2 | 197 | 38.639 | 0.6904 | 37.00 | 5.95E-07 |  |
| **N2 (control)** | 35.8475 | 1.8273 |  | 271 | 35.679 | 0.3971 | 34.00 |  |  |
| **nuo-2 (RNAi)** | 18.8712 | 0.7131 | 5 | 292 | 18.534 | 0.2619 | 18.53 | 4.25E-11 |  |
| **vector (control)** | 19.7298 | 1.5168 |  | 327 | 20.245 | 0.2601 | 20.24 |  |  |
| **phy-2 (RNAi)** | 25.1231 | 0.4956 | 2 | 385 | 25.093 | 0.2488 | 25.09 | 1.11E-11 |  |
| **vector (control)** | 21.6729 | 0.1503 |  | 380 | 21.666 | 0.2537 | 21.67 |  |  |
| **rhy-1 (ok1402)** | 29.9563 | 1.1755 | 5 | 742 | 29.702 | 0.2090 | 29.70 | 0.003236 |  |
| **N2 (control)** | 31.7757 | 1.8517 |  | 704 | 31.563 | 0.2401 | 31.56 |  |  |
| **rol-6 (e187)** | 33.2982 | 1.3944 | 5 | 657 | 33.838 | 0.2750 | 34.00 | 6.76E-14 |  |
| **N2 (control)** | 31.2781 | 1.4965 |  | 619 | 31.368 | 0.2826 | 31.00 |  |  |
| **rpl-6 (RNAi)** | 26.3284 | 1.1704 | 9 | 1166 | 26.327 | 0.1576 | 26.32 | 0.00139 |  |
| **vector (control)** | 28.1362 | 1.2280 |  | 740 | 27.781 | 0.2097 | 27.78 |  |  |
| **rpl-9 (RNAi)** | 19.4252 | 1.0632 | 3 | 284 | 19.350 | 0.2558 | 19.35 | 0.640106 |  |
| **vector (control)** | 22.8971 | 3.4709 |  | 428 | 22.474 | 0.2933 | 22.47 |  |  |
| **rpl-22 (RNAi)** | 21.7975 | 2.0328 | 3 | 460 | 21.093 | 0.2274 | 21.09 | 0.000359 |  |
| **vector (control)** | 23.3280 | 1.6574 |  | 453 | 23.012 | 0.2403 | 23.01 |  |  |
| **rsks-1 (ok1255)** | 27.2270 | 0.0015 | 2 | 238 | 27.226 | 0.3593 | 27.22 | 0.130964 |  |
| **N2 (control)** | 29.7591 | 1.3617 |  | 227 | 30.185 | 0.3346 | 30.19 |  |  |
| **sir-2.1 (ok434)** | 37.3703 | 1.6797 | 5 | 355 | 36.585 | 0.5622 | 36.00 | 4.45E-05 |  |
| **N2 (control)** | 35.5198 | 1.3377 |  | 414 | 35.338 | 0.4272 | 34.00 |  |  |
| **Skn-1 OE (ldIs7)** | 36.1183 | 2.4820 | 5 | 509 | 37.242 | 0.4166 | 37.24 | 6.73E-08 |  |
| ***rol-6* (control)** | 33.2982 | 1.3944 |  | 657 | 33.839 | 0.2751 | 33.84 |  |  |
| **Skn-1 OE (ldIs7)** | 37.3517 | 2.3724 | 6 | 591 | 38.081 | 0.3874 | 38.08 | 4.6E-34 |  |
| **N2 (control)** | 31.8135 | 1.3341 |  | 672 | 31.615 | 0.2680 | 31.61 |  |  |
| **skn-1 (zu67);**  **eu-1** | 26.7325 | 0.8826 | 3 | 520 | 26.738 | 0.2188 | 26.00 | 8.84E-06 |  |
| **N2 (control)** | 29.5709 | 0.8084 |  | 371 | 29.801 | 0.2660 | 30.00 |  |  |
| **skn-1 (zu135);**  **eu-31** | 29.9347 | 4.0856 | 2 | 155 | 28.432 | 0.6360 | 28.43 | 3.82E-07 |  |
| **N2 (control)** | 30.1576 | 0.9632 |  | 293 | 30.174 | 0.3188 | 30.17 |  |  |
| **trpa-1 (ok999)** | 38.1123 | 1.4406 | 2 | 265 | 38.101 | 0.5576 | 34.00 | 0.000907 |  |
| **N2 (control)** | 35.8475 | 1.8273 |  | 271 | 35.679 | 0.3971 | 34.00 |  |  |
| **tyr-2 (ok1363)** | 33.6118 | 0.0855 | 3 | 471 | 33.612 | 0.2395 | 34.00 | 1.31E-12 |  |
| **N2 (control)** | 33.6298 | 2.4558 |  | 415 | 33.429 | 0.3358 | 32.00 |  |  |
| **vhl-1 (ok161)** | 43.0419 | 1.0964 | 21 | 1931 | 43.803 | 0.2106 | 43.80 | 6.5E-152 |  |
| **N2 (control)** | 30.2526 | 0.8404 |  | 2638 | 29.649 | 0.1456 | 29.65 |  |  |
|  | **20oC** |  |  |  |  |  |  |  |  |
| **Genotype/**  **Treatment** | **Averaged Mean** | **Exp. Error** | **Rep** | **N** | **Total Mean** | **Total Error** | **Total Median** | **p-value (compared to N2)** |  |
| **aak-1 (tm1944)** | 23.8208 | 1.5291 | 2 | 88 | 23.681 | 0.5066 | 24.00 | 9.24E-18 |  |
| **N2 (control)** | 31.3283 | 0.4323 |  | 196 | 31.209 | 0.4138 | 32.00 |  |  |
| **aak-2 (ok524)** | 23.7868 | 1.8727 | 5 | 475 | 24.823 | 0.2584 | 25.00 | 0.030651 |  |
| **N2 (control)** | 24.9216 | 1.8616 |  | 663 | 25.881 | 0.2542 | 25.00 |  |  |
| **atm-1 (gk186)** | 21.9566 | 1.3013 | 4 | 414 | 21.978 | 0.2784 | 22.00 | 4.78E-27 |  |
| **N2 (control)** | 29.9752 | 0.8210 |  | 446 | 29.661 | 0.2629 | 30.00 |  |  |
| **atp-3 (RNAi)** | 18.0085 | 0.9263 | 2 | 373 | 18.150 | 0.2015 | 18.15 | 8.22E-16 |  |
| **vector (control)** | 16.0026 | 0.3081 |  | 321 | 16.034 | 0.1773 | 16.03 |  |  |
| **Dietary Restriction (DR)** | 31.6880 | 1.1763 | 8 | 933 | 32.380 | 0.2400 | 32.38 | 5.97E-42 |  |
| **Fed (control)** | 26.3229 | 1.1004 |  | 973 | 26.581 | 0.1968 | 26.58 |  |  |
| **10mM caffeine** | 27.8220 | 2.2451 | 5 | 490 | 27.218 | 0.3143 | 27.21 | 6.33E-07 |  |
| **Water (control)** | 26.7970 | 1.8349 |  | 468 | 26.776 | 0.3187 | 26.78 |  |  |
| **cah-4 (RNAi)** | 17.9257 | 0.8577 | 4 | 508 | 17.537 | 0.1678 | 17.53 | 0.000153 |  |
| **vector (control)** | 17.8276 | 0.5640 |  | 514 | 17.916 | 0.1463 | 17.92 |  |  |
| **cco-1 (RNAi)** | 20.6523 | 2.2853 | 3 | 455 | 19.617 | 0.2395 | 19.61 | 0.036394 |  |
| **vector (control)** | 18.3823 | 2.3863 |  | 434 | 17.885 | 0.2291 | 17.88 |  |  |
| **cep-1 (gk138)** | 26.1478 | 1.2837 | 11 | 1771 | 26.431 | 0.1582 | 27.00 | 3.05E-13 |  |
| **N2 (control)** | 26.2089 | 1.1179 |  | 1637 | 26.276 | 0.1544 | 25.00 |  |  |
| **clk-1 (RNAi)** | 19.0329 | 1.6065 | 3 | 449 | 18.832 | 0.2187 | 18.83 | 1.97E-10 |  |
| **vector (control)** | 18.3823 | 2.3863 |  | 434 | 17.885 | 0.2291 | 17.88 |  |  |
| **cyc-1 (RNAi)** | 21.7346 | 3.5095 | 3 | 477 | 20.454 | 0.3198 | 20.45 | 0.02149 |  |
| **vector (control)** | 18.3823 | 2.3863 |  | 434 | 17.885 | 0.2291 | 17.88 |  |  |
| **daf-2 (e1370)** | 42.6589 | 2.5906 | 5 | 855 | 43.568 | 0.4243 | 46.00 | 1.27E-44 |  |
| **N2 (control)** | 27.9941 | 1.4160 |  | 688 | 27.410 | 0.2108 | 28.00 |  |  |
| **daf-2 (RNAi)** | 33.7672 | 2.3172 | 3 | 187 | 34.550 | 0.7295 | 34.55 | 6.8E-27 |  |
| **vector (control)** | 20.7044 | 1.6324 |  | 168 | 20.815 | 0.3883 | 20.82 |  |  |
| **daf-16 (mu86)** | 20.4089 | 0.8397 | 8 | 693 | 20.537 | 0.1806 | 20.00 | 1.04E-13 |  |
| **N2 (control)** | 23.4754 | 0.9090 |  | 708 | 23.838 | 0.2016 | 24.00 |  |  |
| **egl-9 (n571)** | 28.1178 | 0.6664 | 6 | 1025 | 27.983 | 0.1648 | 28.00 | 0.000335 |  |
| **N2 (control)** | 26.9466 | 0.6803 |  | 829 | 26.423 | 0.1667 | 28.00 |  |  |
| **egl-9 (RNAi)** | 23.7746 | 0.9940 | 3 | 133 | 23.924 | 0.3203 | 23.92 | 4.62E-10 |  |
| **vector (control)** | 20.5868 | 0.9697 |  | 117 | 20.513 | 0.3979 | 20.51 |  |  |
| **egl-9 (sa307)** | 28.0807 | 0.7077 | 7 | 947 | 28.405 | 0.1715 | 29.00 | 2.14E-13 |  |
| **N2 (control)** | 26.4816 | 0.7395 |  | 897 | 26.216 | 0.1617 | 26.00 |  |  |
| **F28B4.5 (RNAi)** | 19.8677 | 0.9179 | 6 | 506 | 18.974 | 0.1683 | 18.97 | 0.078974 |  |
| **vector (control)** | 19.0853 | 0.9075 |  | 558 | 18.398 | 0.1531 | 18.40 |  |  |
| **fmo-2 (RNAi)** | 21.0431 | 1.3273 | 3 | 567 | 20.915 | 0.1818 | 20.91 | 0.629862 |  |
| **vector (control)** | 21.3129 | 1.6868 |  | 510 | 21.614 | 0.1936 | 21.61 |  |  |
| **fmo-2 (ok2147)** | 26.9898 | 1.2637 | 2 | 211 | 27.379 | 0.4512 | 28.00 | 0.012644 |  |
| **N2 (control)** | 26.4896 | 0.0046 |  | 254 | 26.488 | 0.3453 | 26.00 |  |  |
| **gpa-9 (pk438)** | 25.4859 | 0.3256 | 2 | 269 | 25.494 | 0.3052 | 26.00 | 0.360287 |  |
| **N2 (control)** | 26.4896 | 0.0046 |  | 254 | 26.488 | 0.3453 | 26.00 |  |  |
| **hif-1 (ia07)** | 28.5878 | 2.2847 | 4 | 866 | 29.132 | 0.2437 | 29.13 | 6.33E-07 |  |
| **N2 (control)** | 28.4840 | 2.4624 |  | 580 | 28.003 | 0.2806 | 28.00 |  |  |
| **hif-1 (ia04)** | 23.6807 | 0.5972 | 18 | 3197 | 24.827 | 0.1279 | 24.82 | 5.25E-12 |  |
| **N2 (control)** | 23.8882 | 0.5073 |  | 3058 | 24.494 | 0.1043 | 24.49 |  |  |
| **hif-1 (RNAi)** | 20.6582 | 0.8761 | 23 | 2583 | 20.494 | 0.1189 | 20.49 | 5.88E-46 |  |
| **vector (control)** | 19.9860 | 0.6417 |  | 2584 | 19.674 | 0.0967 | 19.67 |  |  |
| **hypoxia (.5%)** | 26.6371 | 1.3099 | 9 | 1104 | 28.028 | 0.1962 | 28.00 | 0.0007 |  |
| **normoxia (21%)** | 23.8261 | 1.1441 |  | 1255 | 25.360 | 0.1551 | 25.00 |  |  |
| **inx-2 (ok376)** | 29.8138 | 0.6271 | 2 | 277 | 29.761 | 0.2853 | 30.00 | 2.99E-06 |  |
| **N2 (control)** | 26.4896 | 0.0046 |  | 254 | 26.488 | 0.3453 | 26.00 |  |  |
| **lys-8 (ok3504)** | 26.0446 | 0.6066 | 2 | 273 | 26.113 | 0.3250 | 26.00 | 0.782727 |  |
| **N2 (control)** | 26.4896 | 0.0046 |  | 254 | 26.488 | 0.3453 | 26.00 |  |  |
| **nuo-2 (RNAi)** | 17.7009 | 1.9262 | 4 | 557 | 17.007 | 0.1969 | 17.00 | 4.36E-13 |  |
| **vector (control)** | 17.9691 | 1.7372 |  | 556 | 17.631 | 0.1879 | 17.63 |  |  |
| **phy-2 (RNAi)** | 20.8526 | 1.9487 | 3 | 132 | 21.030 | 0.3997 | 21.03 | 0.01154 |  |
| **vector (control)** | 20.5868 | 0.9697 |  | 117 | 20.513 | 0.3979 | 20.51 |  |  |
| **rhy-1 (ok1402)** | 23.0575 | 0.9883 | 6 | 915 | 22.893 | 0.1824 | 22.89 | 2.53E-09 |  |
| **N2 (control)** | 27.8723 | 1.1852 |  | 825 | 27.202 | 0.2115 | 27.20 |  |  |
| **rol-6 (e187)** | 25.7377 | 1.5856 | 3 | 427 | 25.339 | 0.3120 | 26.00 | 1.06E-05 |  |
| **N2 (control)** | 27.0531 | 0.5635 |  | 415 | 27.145 | 0.2646 | 28.00 |  |  |
| **rpl-6 (RNAi)** | 21.0707 | 0.7266 | 8 | 756 | 21.247 | 0.1516 | 21.24 | 0.315851 |  |
| **vector (control)** | 22.3337 | 1.0605 |  | 521 | 21.904 | 0.2026 | 21.90 |  |  |
| **rpl-9 (RNAi)** | 20.2547 | 0.3132 | 4 | 551 | 20.157 | 0.1832 | 20.15 | 0.3104 |  |
| **vector (control)** | 21.3416 | 0.9052 |  | 480 | 20.856 | 0.2016 | 20.86 |  |  |
| **rpl-22 (RNAi)** | 21.0665 | 0.4999 | 5 | 583 | 20.842 | 0.1714 | 20.84 | 0.014073 |  |
| **vector (control)** | 20.2764 | 0.4532 |  | 589 | 20.545 | 0.1737 | 20.54 |  |  |
| **rsks-1 (ok1255)** | 26.2212 | 2.1121 | 2 | 206 | 26.077 | 0.3738 | 26.07 | 2.98E-11 |  |
| **N2 (control)** | 25.7932 | 2.3869 |  | 257 | 26.397 | 0.3421 | 26.40 |  |  |
| **sir-2.1 (ok434)** | 24.9069 | 0.9283 | 8 | 923 | 25.109 | 0.1583 | 26.00 | 5.4E-36 |  |
| **N2 (control)** | 26.8938 | 1.2386 |  | 1098 | 27.287 | 0.1961 | 28.00 |  |  |
| **Skn-1 OE (ldIs7)** | 26.9298 | 0.0560 | 3 | 430 | 26.925 | 0.2554 | 26.92 | 2.74E-06 |  |
| ***rol-6* (control)** | 25.7377 | 1.5857 |  | 427 | 25.340 | 0.3120 | 25.34 |  |  |
| **Skn-1 OE (ldIs7)** | 25.6626 | 0.8889 | 11 | 1837 | 25.978 | 0.1422 | 25.97 | 2.42E-05 |  |
| **N2 (control)** | 26.5888 | 1.0349 |  | 1448 | 27.054 | 0.1635 | 27.05 |  |  |
| **skn-1 (zu67);**  **eu-1** | 24.8495 | 1.5813 | 7 | 963 | 24.689 | 0.2004 | 23.00 | 7.86E-05 |  |
| **N2 (control)** | 25.0290 | 1.5262 |  | 993 | 24.863 | 0.1873 | 24.00 |  |  |
| **skn-1 (zu135);**  **eu-31** | 21.4625 | 1.6786 | 6 | 767 | 22.166 | 0.2009 | 22.16 | 3.81E-27 |  |
| **N2 (control)** | 24.5876 | 1.4989 |  | 953 | 25.163 | 0.1939 | 25.16 |  |  |
| **trpa-1 (ok999)** | 26.9034 | 0.1034 | 2 | 278 | 26.906 | 0.3624 | 28.00 | 0.535441 |  |
| **N2 (control)** | 26.4896 | 0.0046 |  | 254 | 26.488 | 0.3453 | 26.00 |  |  |
| **tyr-2 (ok1363)** | 22.4808 | 1.1152 | 3 | 379 | 22.153­­ | 0.2141 | 23.00 | 4.81E-07 |  |
| **N2 (control)** | 24.8820 | 1.6077 |  | 440 | 24.450 | 0.2421 | 23.00 |  |  |
| **vhl-1 (ok161)** | 30.8891 | 0.7414 | 28 | 2931 | 29.946 | 0.1198 | 29.94 | 9.48E-24 |  |
| **N2 (control)** | 23.8943 | 0.5298 |  | 2837 | 24.015 | 0.1082 | 24.01 |  |  |
|  | **25oC** |  |  |  |  |  |  |  |  |
| **Genotype/**  **Treatment** | **Averaged Mean** | **Exp.**  **Error** | **Rep** | **N** | **Total Mean** | **Total Error** | **Total Median** | **p-value (compared to N2)** |  |
| **aak-1 (tm1944)** | 14.4258 | 0.5179 | 2 | 330 | 14.466 | 0.1801 | 14.00 | 2.51E-06 |  |
| **N2 (control)** | 15.8656 | 0.6957 |  | 308 | 15.870 | 0.1852 | 16.00 |  |  |
| **aak-2 (ok524)** | 14.6095 | 0.7878 | 2 | 280 | 14.671 | 0.1394 | 14.00 | 0.000138 |  |
| **N2 (control)** | 15.8656 | 0.6957 |  | 308 | 15.870 | 0.1852 | 16.00 |  |  |
| **atm-1 (gk186)** | 16.7794 | 0.9669 | 2 | 212 | 16.451 | 0.2943 | 16.00 | 0.147462 |  |
| **N2 (control)** | 15.8656 | 0.6957 |  | 308 | 15.870 | 0.1852 | 16.00 |  |  |
| **atp-3 (RNAi)** | 15.3811 | 0.9662 | 4 | 644 | 15.184 | 0.1381 | 15.18 | 0.019101 |  |
| **vector (control)** | 14.2439 | 1.0018 |  | 721 | 14.311 | 0.1041 | 14.31 |  |  |
| **Dietary Restriction (DR)** | 22.0954 | 1.4969 | 2 | 294 | 22.197 | 0.3600 | 22.19 | 1.31E-29 |  |
| **Fed (control)** | 15.8656 | 0.6957 |  | 308 | 15.870 | 0.1852 | 15.87 |  |  |
| **10mM caffeine** | 17.3554 | 3.7614 | 2 | 178 | 16.848 | 0.3876 | 16.84 | 1.07E-08 |  |
| **Water (control)** | 19.3120 | 2.9787 |  | 170 | 19.347 | 0.3769 | 19.35 |  |  |
| **cah-4 (RNAi)** | 14.4023 | 0.0750 | 2 | 320 | 14.400 | 0.1173 | 14.40 | 0.000748 |  |
| **vector (control)** | 15.1763 | 0.0375 |  | 289 | 15.176 | 0.1516 | 15.18 |  |  |
| **cco-1 (RNAi)** | 15.4544 | 0.7142 | 4 | 566 | 15.286 | 0.1641 | 15.28 | 0.001286 |  |
| **vector (control)** | 13.4137 | 0.8896 |  | 638 | 13.806 | 0.1073 | 13.81 |  |  |
| **cep-1 (gk138)** | 17.1625 | 1.5704 | 3 | 453 | 16.966 | 0.1859 | 17.00 | 0.746163 |  |
| **N2 (control)** | 16.4714 | 0.7269 |  | 450 | 16.442 | 0.1499 | 17.00 |  |  |
| **clk-1 (RNAi)** | 13.5173 | 1.2627 | 5 | 702 | 13.377 | 0.1334 | 13.37 | 0.057545 |  |
| **vector (control)** | 13.9054 | 0.8466 |  | 779 | 14.180 | 0.0989 | 14.18 |  |  |
| **cyc-1 (RNAi)** | 14.8101 | 0.8309 | 4 | 563 | 14.879 | 0.1586 | 14.87 | 0.108162 |  |
| **vector (control)** | 13.4137 | 0.8896 |  | 638 | 13.806 | 0.1073 | 13.81 |  |  |
| **daf-2 (e1370)** | 35.3257 | 1.8097 | 2 | 312 | 35.314 | 0.4163 | 35.00 | 2.2E-50 |  |
| **N2 (control)** | 21.9517 | 0.9225 |  | 296 | 22.020 | 0.1843 | 22.00 |  |  |
| **daf-2 (RNAi)** | 26.6356 | 0.8765 | 3 | 240 | 25.895 | 0.4534 | 25.89 | 3.31E-42 |  |
| **vector (control)** | 14.8038 | 0.3731 |  | 306 | 15.114 | 0.1487 | 15.11 |  |  |
| **daf-16 (mu86)** | 15.3958 | 0.5247 | 4 | 315 | 15.200 | 0.1275 | 15.00 | 5.79E-16 |  |
| **N2 (control)** | 18.5590 | 0.5685 |  | 277 | 18.553 | 0.2077 | 18.00 |  |  |
| **egl-9 (n571)** | 17.6966 | 1.1717 | 4 | 408 | 17.223 | 0.1876 | 17.00 | 0.755425 |  |
| **N2 (control)** | 18.1461 | 1.2199 |  | 576 | 17.866 | 0.1620 | 17.00 |  |  |
| **egl-9 (RNAi)** | 16.5142 | 0.5918 | 4 | 276 | 16.174 | 0.1305 | 16.17 | 0.00043 |  |
| **vector (control)** | 16.0013 | 0.7256 |  | 380 | 15.632 | 0.1344 | 15.63 |  |  |
| **egl-9 (sa307)** | 19.4073 | 0.5058 | 4 | 572 | 19.141 | 0.1780 | 19.00 | 3.12E-08 |  |
| **N2 (control)** | 18.1461 | 1.2199 |  | 756 | 17.866 | 0.1620 | 17.00 |  |  |
| **F28B4.5 (RNAi)** | 15.0611 | 0.1732 | 3 | 337 | 14.997 | 0.1408 | 14.99 | 0.144644 |  |
| **vector (control)** | 15.2795 | 0.1054 |  | 324 | 15.210 | 0.1414 | 15.21 |  |  |
| **fmo-2 (RNAi)** | 15.6366 | 0.0942 | 6 | 784 | 15.623 | 0.0920 | 15.62 | 0.213267 |  |
| **vector (control)** | 15.9761 | 0.2896 |  | 804 | 15.978 | 0.1066 | 15.98 |  |  |
| **fmo-2 (ok2147)** | 21.3042 | 0.1282 | 2 | 162 | 21.234 | 0.2319 | 21.00 | 0.007665 |  |
| **N2 (control)** | 20.6394 | 0.4763 |  | 288 | 20.649 | 0.1887 | 21.00 |  |  |
| **gpa-9 (pk438)** | 21.0839 | 1.0306 | 2 | 307 | 21.107 | 0.2855 | 21.00 | 8.59E-07 |  |
| **N2 (control)** | 18.4969 | 0.4908 |  | 326 | 18.497 | 0.2263 | 17.00 |  |  |
| **hif-1 (ia07)** | 18.0929 | 0.2007 | 2 | 327 | 18.088 | 0.2209 | 18.08 | 3.18E-12 |  |
| **N2 (control)** | 15.8656 | 0.6957 |  | 308 | 15.870 | 0.1852 | 15.87 |  |  |
| **hif-1 (ia04)** | 20.6809 | 0.2961 | 11 | 2306 | 21.087 | 0.0835 | 21.08 | 9.31E-15 |  |
| **N2 (control)** | 17.8533 | 0.3608 |  | 2123 | 18.646 | 0.0754 | 18.65 |  |  |
| **hif-1 (RNAi)** | 16.4999 | 1.1129 | 8 | 1072 | 16.039 | 0.1280 | 16.03 | 0.874266 |  |
| **vector (control)** | 15.7604 | 0.6042 |  | 1073 | 15.286 | 0.1095 | 15.29 |  |  |
| **hypoxia (.5%)** | 20.8026 | 0.3776 | 3 | 622 | 20.871 | 0.1308 | 21.00 | 3.69E-06 |  |
| **normoxia (21%)** | 18.8602 | 0.5945 |  | 545 | 18.989 | 0.1239 | 18.00 |  |  |
| **inx-2 (ok376)** | 21.2884 | 0.7115 | 2 | 171 | 21.350 | 0.2746 | 22.00 | 0.036735 |  |
| **N2 (control)** | 20.6394 | 0.4763 |  | 288 | 20.649 | 0.1887 | 21.00 |  |  |
| **lys-8 (ok3504)** | 21.2879 | 1.7977 | 2 | 233 | 20.618 | 0.3191 | 21.00 | 4.11E-05 |  |
| **N2 (control)** | 18.4969 | 0.4908 |  | 326 | 18.497 | 0.2263 | 17.00 |  |  |
| **nuo-2 (RNAi)** | 15.4186 | 1.6212 | 2 | 700 | 15.865 | 0.1728 | 15.86 | 4.57E-09 |  |
| **vector (control)** | 13.9054 | 0.8466 |  | 779 | 14.180 | 0.0989 | 14.18 |  |  |
| **phy-2 (RNAi)** | 16.8561 | 1.6532 | 2 | 121 | 16.639 | 0.2463 | 16.63 | 0.57281 |  |
| **vector (control)** | 16.8262 | 1.3405 |  | 89 | 17.112 | 0.2285 | 17.11 |  |  |
| **rhy-1 (ok1402)** | 14.6166 | 0.8027 | 5 | 980 | 14.070 | 0.1037 | 14.07 | 1.02E-56 |  |
| **N2 (control)** | 17.0406 | 0.6622 |  | 839 | 17.039 | 0.1242 | 17.04 |  |  |
| **rol-6 (e187)** | 33.2982 | 1.3944 | 5 | 679 | 33.838 | 0.2750 | 34.00 | 0.004824 |  |
| **N2 (control)** | 17.5917 | 0.7663 |  | 771 | 17.578 | 0.1393 | 17.00 |  |  |
| **rpl-6 (RNAi)** | 18.2221 | 0.2895 | 4 | 461 | 18.199 | 0.1658 | 18.19 | 0.003761 |  |
| **vector (control)** | 15.6325 | 0.5989 |  | 405 | 16.054 | 0.1924 | 16.05 |  |  |
| **rpl-9 (RNAi)** | 16.6971 | 0.6527 | 3 | 391 | 16.304 | 0.1345 | 16.30 | 0.003597 |  |
| **vector (control)** | 14.8038 | 0.3731 |  | 306 | 15.114 | 0.1487 | 15.11 |  |  |
| **rpl-22 (RNAi)** | 15.5370 | 0.4171 | 3 | 281 | 15.561 | 0.1428 | 15.56 | 0.07451 |  |
| **vector (control)** | 15.6867 | 0.5108 |  | 521 | 15.731 | 0.1637 | 15.73 |  |  |
| **rsks-1 (ok1255)** | 21.8313 | 1.4795 | 4 | 574 | 20.588 | 0.1868 | 20.58 | 2.03E-12 |  |
| **N2 (control)** | 17.6969 | 0.5400 |  | 646 | 17.704 | 0.1442 | 17.70 |  |  |
| **sir-2.1 (ok434)** | 16.8612 | 0.8868 | 4 | 647 | 16.589 | 0.1439 | 16.00 | 5.63E-11 |  |
| **N2 (control)** | 16.3812 | 0.4554 |  | 628 | 16.393 | 0.1259 | 16.00 |  |  |
| **Skn-1 OE (ldIs7)** | 14.7445 | 0.9253 | 3 | 402 | 14.380 | 0.1186 | 14.38 | 0.139615 |  |
| ***rol-6* (control)** | 15.3006 | 1.1411 |  | 383 | 15.504 | 0.1581 | 15.50 |  |  |
| **Skn-1 OE (ldIs7)** | 14.7445 | 0.9253 | 3 | 402 | 14.380 | 0.1186 | 14.38 | 6.98E-08 |  |
| **N2 (control)** | 16.9883 | 1.1923 |  | 445 | 16.906 | 0.1687 | 16.91 |  |  |
| **skn-1 (zu67);**  **eu-1** | 16.2139 | 0.8022 | 3 | 502 | 16.069 | 0.1439 | 16.00 | 0.806487 |  |
| **N2 (control)** | 16.9883 | 1.1923 |  | 445 | 16.906 | 0.1687 | 17.00 |  |  |
| **skn-1 (zu135);**  **eu-31** | 18.2702 | 0.5827 | 2 | 316 | 18.439 | 0.1784 | 18.43 | 6.25E-05 |  |
| **N2 (control)** | 18.3412 | 0.8924 |  | 264 | 18.375 | 0.1940 | 18.38 |  |  |
| **trpa-1 (ok999)** | 21.4479 | 0.3597 | 2 | 172 | 21.523 | 0.3710 | 21.00 | 2.12E-05 |  |
| **N2 (control)** | 18.4969 | 0.4908 |  | 326 | 18.497 | 0.2263 | 17.00 |  |  |
| **tyr-2 (ok1363)** | 18.7894 | 1.4156 | 2 | 138 | 18.173 | 0.3258 | 17.00 | 0.084695 |  |
| **N2 (control)** | 18.4969 | 0.4908 |  | 326 | 18.497 | 0.2263 | 17.00 |  |  |
| **vhl-1 (ok161)** | 23.5363 | 0.5355 | 22 | 1737 | 23.667 | 0.1172 | 23.66 | 1.11E-23 |  |
| **N2 (control)** | 18.3044 | 0.4195 |  | 1901 | 18.567 | 0.0930 | 18.57 |  |  |

Column keys: averaged mean = average lifespan across replicate experiments, experimental error = standard error across replicate experiments, rep = number of replicate experiments, n = animals included in all replicate experiments, total mean = average combining replicate experiments, total error = standard error combining replicate experiments, total Median = median combining replicate experiments, p-value (compared to N2) = students two-tailed t-test.

**Supplemental Table 3. Hazard Ratio calculations for Figure 2C-D, Figure S6.**

| **condition** | **Hazard ratio** | **Standard Error** | | | **p-value** | **95% confidence interval** | |
| --- | --- | --- | --- | --- | --- | --- | --- |
| ***aak-2*** | **1.883359** | **0.254816** | | **4.68** | **<0.001** | **1.444663** | **2.455272** |
| ***atm-1*** | **2.423418** | **0.310277** | | **6.91** | **<0.001** | **1.885587** | **3.114656** |
| **Dietary Restriction (DR)** | **4.271351** | **0.479679** | | **12.93** | **<0.001** | **3.427473** | **5.323001** |
| ***daf-2*** | **2.628244** | **0.313828** | | **8.09** | **<0.001** | **2.079826** | **3.32127** |
| ***gpa-9*** | **2.013283** | **0.23811** | | **5.92** | **<0.001** | **1.596738** | **2.538494** |
| ***rsks-1*** | **2.961879** | **0.327957** | | **9.81** | **<0.001** | **2.384061** | **3.679742** |
| ***hif-1 ia4*** | **2.005512** | **0.084294** | | **16.56** | **<0.001** | **1.84692** | **2.177721** |
| ***hif-1 ia7*** | **1.405371** | **0.154761** | | **3.09** | **0.002** | **1.132545** | **1.74392** |
| ***trpa-1*** | **1.439773** | **0.188655** | | **2.78** | **0.005** | **1.113679** | **1.86135** |
| ***cep-1*** | **0.685007** | **0.07829** | | **-3.31** | **0.001** | **0.547533** | **0.856998** |
| ***daf-16*** | **0.39232** | **0.039225** | | **-9.36** | **<0.001** | **0.322505** | **0.477249** |
| ***egl-9(n571)*** | **0.416451** | **0.037096** | | **-9.83** | **<0.001** | **0.349737** | **0.495891** |
| **Caffeine** | **0.359499** | **0.043363** | | **-8.48** | **<0.001** | **0.283808** | **0.455375** |
| ***vhl-1*** | **0.726289** | **0.034123** | | **-6.81** | **<0.001** | **0.662396** | **0.796345** |
| ***rol-6*** | **0.463335** | **0.036049** | | **-9.89** | **<0.001** | **0.397804** | **0.539661** |
| ***SKN-1 OX*** | **0.185997** | **0.017197** | | **-18.19** | **<0.001** | **0.155168** | **0.222951** |
| ***SKN-1 Oxb*** | **0.394067** | **0.037374** | | **-9.82** | **<0.001** | **0.327221** | **0.474568** |
| ***inx-2*** | **0.678473** | **0.082781** | | **-3.18** | **0.001** | **0.534168** | **0.861763** |
| ***rhy-1*** | **0.571705** | **0.04146** | | **-7.71** | **<0.001** | **0.495955** | **0.659023** |
| ***fmo-2*** | **1.011335** | **0.139118** | | **0.08** | **0.935** | **0.772334** | **1.324296** |
| ***egl-9(sa307)*** | **0.863113** | **0.07188** | | **-1.77** | **0.077** | **0.733127** | **1.016145** |
| ***aak-1*** | **0.868319** | **0.099977** | | **-1.23** | **0.22** | **0.692904** | **1.088141** |
| **Hypoxia (0.5% O_2_)** | **0.904899** | **0.088328** | | **-1.02** | **0.306** | **0.747333** | **1.095687** |
| ***FMO-2 OX*** | **0.933723** | **0.087696** | | **-0.73** | **0.465** | **0.776735** | **1.122441** |
| ***tyr-2*** | **0.978058** | **0.119694** | | **-0.18** | **0.856** | **0.769476** | **1.243181** |
| ***skn-1 zu135*** | **1.1333** | **0.148722** | | **0.95** | **0.34** | **0.87628** | **1.465706** |
| ***skn-1 zu67*** | **1.109203** | **0.104894** | | **1.1** | **0.273** | **0.921543** | **1.335079** |
| ***sir2.1*** | **1.01132** | **0.093278** | | **0.12** | **0.903** | **0.84407** | **1.211709** |
| ***lys-8*** | **1.147747** | **0.151666** | | **1.04** | **0.297** | **0.885863** | **1.487053** |
| **RNAi** | **Hazard ratio** | **Standard Error** | | | **p-value** | **95% confidence interval** | |
| ***daf-2*** | **2.464961** | **0.342478** | **6.49** | | **<0.001** | **1.877351** | **3.236492** |
| ***cco-1*** | **1.469224** | **0.135519** | **4.17** | | **<0.001** | **1.226237** | **1.760361** |
| ***rpl-9*** | **2.84442** | **0.314401** | **9.46** | | **<0.001** | **2.290383** | **3.532477** |
| ***rpl-6*** | **1.851258** | **0.15362** | **7.42** | | **<0.001** | **1.573379** | **2.178215** |
| ***nuo-2*** | **2.318233** | **0.226252** | **8.62** | | **<0.001** | **1.91462** | **2.80693** |
| ***hif-1*** | **1.517506** | **0.092637** | **6.83** | | **<0.001** | **1.346382** | **1.710381** |
| ***cah-4*** | **0.639755** | **0.069733** | **-4.1** | | **<0.001** | **0.516695** | **0.792125** |
| ***clk-1*** | **0.770578** | **0.069769** | **-2.88** | | **0.004** | **0.645279** | **0.920206** |
| ***egl-9*** | **0.35076** | **0.041811** | **-8.79** | | **<0.001** | **0.27768** | **0.443073** |
| ***phy-2*** | **0.549394** | **0.086731** | **-3.79** | | **<0.001** | **0.403189** | **0.748617** |
| ***atp-3*** | **0.928429** | **0.086832** | **-0.79** | | **0.427** | **0.772929** | **1.115213** |
| ***cyc-1*** | **1.167516** | **0.110711** | **1.63** | | **0.102** | **0.969498** | **1.40598** |
| ***F28B5.4*** | **0.949464** | **0.099172** | **-0.5** | | **0.62** | **0.773695** | **1.165163** |
| ***fmo-2*** | **0.954942** | **0.075838** | **-0.58** | | **0.562** | **0.817292** | **1.115775** |
